# Wheat Cultivation Suitability Evaluation with Stripe Rust Disease: An Agricultural Group Consensus Framework Based on Artificial-Intelligence-Generated Content and Optimization-Driven Overlapping Community Detection

**DOI:** 10.3390/plants14121794

**Published:** 2025-06-11

**Authors:** Tingyu Xu, Haowei Cui, Yunsheng Song, Chao Zhang, Turki Alghamdi, Majed Aborokbah

**Affiliations:** 1 School of Computer and Information Technology, Shanxi University, Taiyuan 030006, China; xutingyu@sxu.edu.cn (T.X.); cuihaowei@sxu.edu.cn (H.C.); 2 College of Information Science and Engineering, Shandong Agricultural University, Taian 271018, China; songys@sdau.edu.cn; 3 Faculty of Computer, Islamic University of Madinah, Madinah 42351, Saudi Arabia; dr.turki2@iu.edu.sa; 4 Faculty of Computers and Information Technology, University of Tabuk, Tabuk 71491, Saudi Arabia; m.aborokbah@ut.edu.sa

**Keywords:** precision agriculture, wheat cultivation, wheat strip rust disease, artificial intelligent generated content, plant disease detection

## Abstract

Plant modeling uses mathematical and computational methods to simulate plant structures, physiological processes, and interactions with various environments. In precision agriculture, it enables the digital monitoring and prediction of crop growth, supporting better management and efficient resource use. Wheat, as a major global staple, is vital for food security. However, wheat stripe rust, a widespread and destructive disease, threatens yield stability. The paper proposes wheat cultivation suitability evaluation with stripe rust disease using an agriculture group consensus framework (WCSE-AGC) to tackle this issue. Assessing stripe rust severity in regions relies on wheat pathologists’ judgments based on multiple criteria, creating a multi-attribute, multi-decision-maker consensus problem. Limited regional coverage and inconsistent evaluations among wheat pathologists complicate consensus-reaching. To support wheat pathologist participation, this study employs artificial-intelligence-generated content (AIGC) techniques by using Claude 3.7 to simulate wheat pathologists’ scoring through role-playing and chain-of-thought prompting. WCSE-AGC comprises three main stages. First, a graph neural network (GNN) models trust propagation within wheat pathologists’ social networks, completing missing trust links and providing a solid foundation for weighting and clustering. This ensures reliable expert influence estimations. Second, integrating secretary bird optimization (SBO), K-means, and three-way clustering detects overlapping wheat pathologist subgroups, reducing opinion divergence and improving consensus inclusiveness and convergence. Third, a two-stage optimization balances group fairness and adjustment cost, enhancing consensus practicality and acceptance. The paper conducts experiments using publicly available real wheat stripe rust datasets from four different locations, Ethiopia, India, Turkey, and China, and validates the effectiveness and robustness of the framework through comparative and sensitivity analyses.

## 1. Introduction

In the wave of agricultural digitalization, plant modeling has emerged as a core approach driving industry transformation due to its unique advantages. As an interdisciplinary method combining modern agricultural science and computational modeling, plant modeling uses mathematical formulations and computer simulations to represent the full life cycle of plant morphological developments, physiological processes, and complex interactions with environmental factors [1]. This transforms plants from tangible living organisms into precisely analyzable digital models, enabling the visualization and quantification of biological processes [2]. By relying on these models, researchers can gain deeper insights into plant responses to environmental stress, predict growth dynamics under varying conditions, and provide quantitative support for decision-making in agricultural practices [3]. Ultimately, plant modeling facilitates the shift from experience-based to data-driven agricultural management paradigms [4,5].

Precision agriculture [6], as a data-centric paradigm of modern agricultural management, is built upon the foundation of information technology and data analytics, aiming to achieve the refined and intelligent control of production processes [7]. In this context, plant modeling plays an indispensable role [8]. By enabling real-time digital monitoring of plant growth and the dynamic prediction of development trajectories, it allows for the optimization of cultivation plans [9] and disease control strategies [10] via the integration of multidimensional data such as climate and soil properties. Consequently, plant modeling empowers farmers to make informed decisions that ensure high-quality, high-yield crop production while minimizing resource waste and environmental impact.

Taking wheat cultivation as a representative case, this staple crop is essential to global food security [11]. However, wheat stripe rust, a highly prevalent and destructive disease, poses a major threat to wheat yield and quality, undermining the stability of food supply chains [12]. To address this pressing issue, this study incorporates publicly available and authentic wheat stripe rust datasets from four countries, Ethiopia, India, Turkey, and China [13], and integrates plant modeling techniques with expert consensus approaches. Through this framework, this paper systematically evaluates the suitability of wheat cultivation under the threat of stripe rust, providing robust scientific support for precision agriculture practices and contributing to the sustainable development of the wheat industry.

### 1.1. The Wheat Stripe Rust Evaluation Framed as a WCSE-AGC

**Figure 1:**
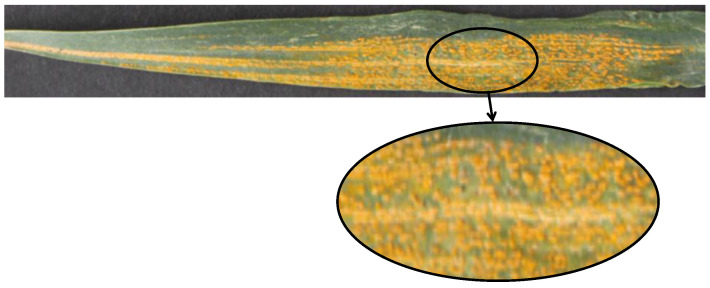
The visualization of wheat stripe rust infection status.

Wheat, a vital crop, serves as the primary food source for billions, especially in developing nations, and plays a central role in agriculture. Consequently, wheat diseases, particularly wheat rust, have attracted significant research attention, with advancements in detection and prevention [14,15]. Wheat rust, a severe fungal disease, threatens wheat production, progressing through various stages [16]. Initially, pale yellow to orange-yellow streaks appear on wheat leaves, running parallel to leaf veins. As the disease advances, these streaks expand and merge into yellow bands. In severe cases, the entire leaf turns yellow and necrotic, with orange-yellow spores that may spread to the spikes and stems [17]. The detailed disease status of wheat leaf rust is illustrated in Figure 1. If untreated, wheat rust can reduce photosynthetic efficiency, causing yield losses of 30–100% and decrease grain quality and plant resilience to stressors, ultimately threatening farmer incomes and food security [18,19]. Early and accurate assessment is essential for effective control [20].

**Figure 2:**
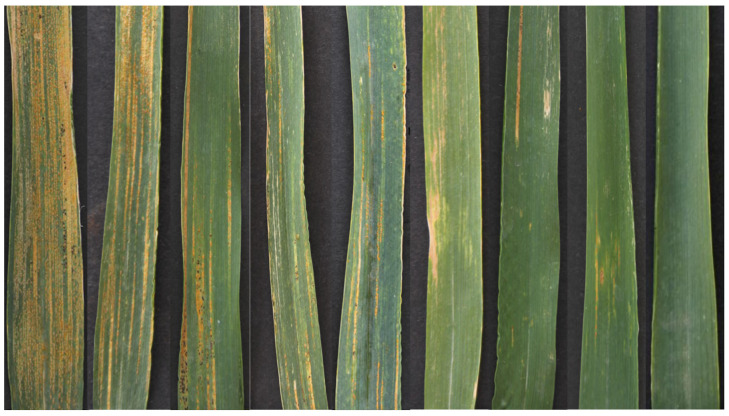
Comparison of wheat leaf rust severity at different infection levels.

The severity of wheat stripe rust in a specific region is typically assessed based on multiple key factors, such as infection area ratio, lesion density, spore production level, and lesion color vividness. These factors need to be considered comprehensively. Wheat pathologists generally rely on these indicators to assess the distribution, extent of infection, and progression of the disease. The specific comparison of different severity levels of wheat leaf rust is illustrated in Figure 2. However, in practical applications, different wheat pathologists often provide varying evaluations of disease severity in the same region due to differences in their knowledge structures, accumulated experience, and perceptual biases. This subjectivity and inconsistency are particularly pronounced in large-scale disease monitoring, potentially leading to increased uncertainty in the evaluation results, which may, in turn, affect the scientific accuracy and effectiveness of control strategies.

In light of these challenges, modeling the detection and assessment of wheat stripe rust as a group consensus problem is of considerable practical significance. In this framework, multiple wheat pathologists can provide individual judgments based on the aforementioned evaluation indicators. The system integrates these diverse opinions, assesses the degree of consistency among them, and uses consensus adjustment mechanisms to resolve significant discrepancies, ultimately producing a collective evaluation with strong representativeness and minimal bias. This approach not only helps mitigate the uncertainty introduced by individual judgments but also enhances the stability, objectivity, and interpretability of overall assessments.

### 1.2. The Simulation of Wheat Pathologists for Evaluating Wheat Cultivations

**Figure 3:**
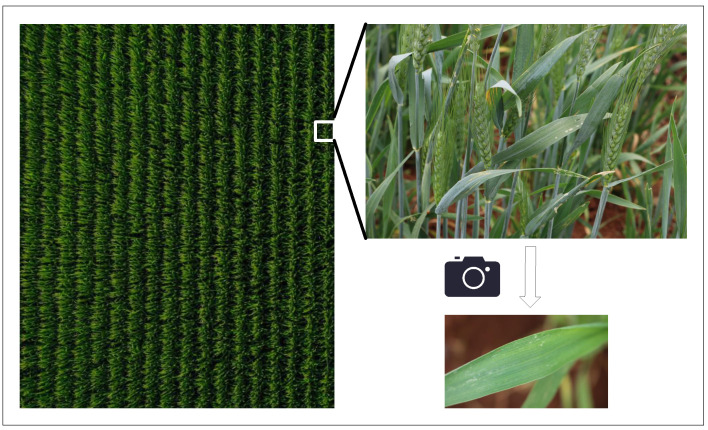
Display of data collection methods.

**Figure 4:**
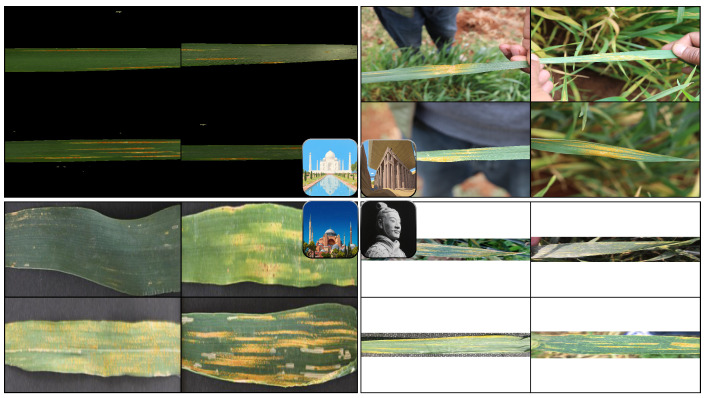
Overview of relevant agricultural datasets.

This study is based on a publicly available dataset, where all images were collected through standardized manual field photography. As shown in Figure 3, the rigorous acquisition process ensures data integrity and traceability. Figure 4 displays representative wheat leaf rust samples, which form the core for assessing disease severity.

However, scoring such images relies heavily on wheat pathologists’ judgment in plant pathology. Public datasets are limited by the uneven geographic distribution of wheat pathologists and the time-consuming nature of manual annotation. These challenges lead to sparse, unbalanced data, restricting the generalizability and depth of research findings. Moreover, the complexity of rust severity assessment, marked by diverse visual features, makes it difficult for automated systems to fully capture disease characteristics. Multi-expert scoring can help mitigate subjectivity and incorporate diverse domain knowledge.

**Figure 5:**
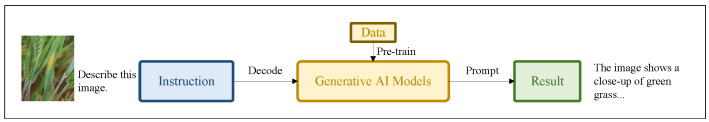
Workflow diagram of image content processing in agricultural applications using chain-of-thought reasoning with AIGC.

To overcome these limitations, this paper constructs an innovative augmentation approach using AIGC, as illustrated in Figure 5. AIGC, powered by large-scale pretrained models, can generate coherent and high-quality labels without relying on handcrafted rules. This enables efficient, scalable, and adaptable annotation, particularly suitable for high-cost wheat pathologist tasks.

This paper employs Claude 3.7 as the core model, integrating chain-of-thought prompting and role-playing strategies to simulate wheat pathologist scoring.

**Figure 6:**
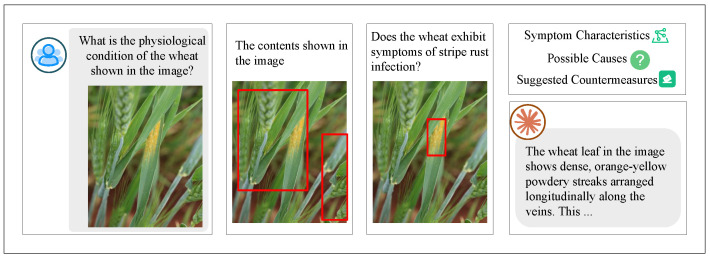
Workflow diagram of image content processing in agricultural applications using chain-of-thought reasoning with AIGC.

As illustrated in Figure 6, the chain-of-thought strategy guides the model through step-by-step reasoning, enabling a structured analysis of disease traits. This approach enhances both the accuracy and interpretability of the generated scores by mimicking wheat pathologists’ analytical processes.

**Figure 7:**
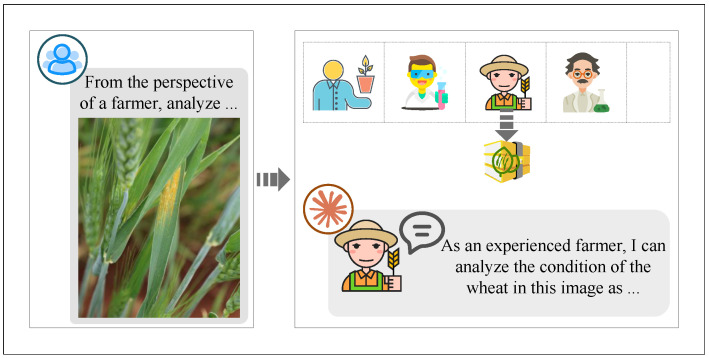
Workflow diagram of wheat condition image processing using role-playing and AIGC techniques.

As illustrated in Figure 7, the role-playing strategy assigns the model diverse wheat pathologist personas with varying backgrounds and perspectives, simulating a multi-expert evaluation process. This approach captures a broader range of judgment criteria, making the scores more realistic and nuanced.

AIGC-generated pseudo-labels fill regional data gaps, enhance sample diversity, and simulate inter-expert variability. Compared to traditional expert-only annotations, this method significantly reduces costs and improves efficiency while providing a robust and representative dataset for downstream consensus modeling and precise disease assessment, advancing the practical deployment of intelligent agricultural diagnostics.

### 1.3. Trust Propagation Among Wheat Pathologists’ Wheat Rust Ratings

In the WCSE-AGC model, wheat pathologists’ expert judgments form the foundation of decision-making. However, due to differences in knowledge structures, research focuses, and practical experience, divergent opinions often arise in assessing disease severity or control priorities. Without effective trust modeling and propagation, communication can break down, leading to isolated knowledge silos and opinion polarization, which undermine the scientific validity of group decisions. Building a trust propagation model enables the quantification of inter-expert trusts, addresses evaluation gaps, and improves credibility in opinion exchanges, providing a solid basis for accurate and efficient disease control decisions.

Research on trust matrix completion originated in social network analysis (SNA) to address incomplete data. Early methods, such as mean or median imputation, overlooked intrinsic network structures, limiting accuracy. Candes and Recht [21] enhanced this by proposing nuclear norm minimization, significantly improving accuracy; however, these methods generally ignore network-specific transitive properties, which are crucial in social trust.

To address this, Pavleska [22] incorporated trust propagation into matrix completion, considering transitive relationships within the network. This advancement better reflects social trust dynamics but still relies on static network assumptions and lacks adaptability to changing expert relationships over time. More recently, Wang et al. [23] leveraged attention mechanisms combined with graph convolutional networks (GCNs) to perform trust matrix completion with uncertainty awareness, offering improved flexibility and context sensitivity. Nonetheless, such deep learning approaches require large datasets and can be computationally intensive, which may limit their applicability in real-time or resource-constrained agricultural decision-making environments.

Furthermore, while progress has been made, existing approaches generally lack interpretability, making it difficult to understand the rationale behind completed trust values. They also insufficiently address inverse trust relationships and the varying impact of different trust propagation paths on final outcomes. By tailoring trust propagation and completion mechanisms to the WCSE-AGC framework, it is possible to enhance decision-making efficiency and accuracy in wheat stripe rust control, while better handling expert opinion divergence and incomplete information for a more robust and realistic consensus reaching process (CRP).

### 1.4. Multi-Group Wheat Pathologist Detection

In WCSE-AGC-based large-scale group decision-making (LSGDM) for wheat stripe rust control, differences in knowledge background, research focus, and practical experience among wheat pathologists often impede effective collaboration and information flow. To overcome these challenges, overlapping community detection is utilized to identify expert subgroups with shared or intersecting expertise, facilitating cross-domain knowledge integration and complementarity in disease diagnosis and strategy formulation. This enhances the diversity, comprehensiveness, and scientific rigor of the decision-making process.

Traditional clustering algorithms such as K-means and hierarchical clustering [24] have long been used for community detection but assume exclusive membership, limiting their ability to capture the multifaceted nature of expert networks. Modularity-based methods introduced by Newman [25] gave rise to Louvain and Leiden algorithms, which improve detection efficiency and incorporate trust dynamics [26,27], but still produce non-overlapping communities.

Recognizing that experts often belong to multiple overlapping groups, Palla et al. [28] proposed the CFinder algorithm based on k-clique percolation, pioneering overlapping community detection. Ji et al. [29] further applied this concept to design feedback mechanisms within expert communities to optimize CRP, though computational costs remain high in large-scale settings. To address efficiency, Teng et al. [30] enhanced the speaker–listener label propagation algorithm with weighted directed strategies, improving accuracy but facing scalability and convergence challenges as the network size grows.

The WCSE-AGC framework integrates a lightweight, multi-criteria fusion approach for overlapping community detections, balancing multiple clustering metrics with methodological simplicity and low computational overhead. This enables the practical, scalable identification of expert subgroups in complex wheat pathologist networks, ultimately supporting more adaptive and scientifically grounded decisions for wheat stripe rust management.

### 1.5. The Fairness and Cost-Aware Wheat Pathologist Opinion Adjustments

In the WCSE-AGC mechanism for wheat stripe rust control, fairness perception and adjustment cost are two critical factors that influence wheat pathologists’ decision behaviors. Integrating both into the CRP allows for more realistic modeling of expert psychology and practical constraints, leading to more reliable and efficient decision-making [31,32].

Fairness perception helps ensure balanced participation and reduces bias caused by dominant opinions, fostering cooperation. Meanwhile, adjustment costs reflect the effort required for opinion modification, which is crucial for optimizing consensus efficiency and resource allocation. The joint consideration of these two aspects enables fair yet cost-effective CRP.

Prior studies have highlighted the importance of fairness. Fehr and Schmidt [33] showed that individuals often prioritize fairness over personal gain. Liu et al. [34] and Ernst et al. [35] emphasized its role in participation and sustainable outcomes. Starke et al. [36] identified fairness as a context-dependent, multi-dimensional concept, while Jiang et al. [37] stressed transparency and human agency in artificial intelligence (AI) involved decisions. Li et al. [38] provided neural evidence of fairness sensitivity in large groups.

However, many current models overlook adjustment costs, making them impractical in real-world applications. They often use single consensus metrics, lack robustness to environmental changes, and ignore the heterogeneity of expert costs and trust structures.

In non-optimization-based CRP, Ding et al. [39,40] introduced confidence and conflict metrics to guide opinion adjustment, but their effectiveness diminishes in large-scale, nonlinear networks. In optimization-based CRP, Song et al. [41] and Zhou et al. [42] used trust-based models and optimization to reduce consensus cost. However, they struggled with scalability, adaptability, and differentiation of individual expert constraints.

To overcome these issues, we propose a fairness- and cost-aware optimization framework within WCSE-AGC. By integrating dynamic fairness modeling, trust-based opinion representation, and refined cost control, this method supports robust, scalable, and realistic expert consensus in complex disease management contexts.

### 1.6. Motivations and Innovations of the Paper

**Figure 8:**
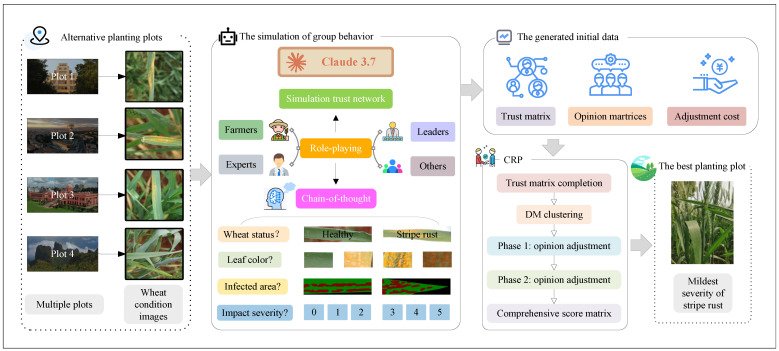
Overall framework for selecting wheat stripe rust disease symptoms with the least severity for planting locations.

While simulating the process of wheat pathologist scoring, the model also simulates the trust relationships between wheat pathologists and the cost of adjusting their opinions. Through the data provided during the simulation, wheat pathologist clustering and opinion adjustment are performed to achieve consensus. The framework for this process is illustrated in Figure 8.

Real-world agricultural decision-making faces challenges such as limited wheat pathologist availability, uncertain trust relationships, diverse wheat pathologist backgrounds, and the need to balance fairness with adjustment cost. This study is motivated by the following:

(1) Limited wheat pathologist access. In many regions, especially remote areas, wheat pathologists are scarce. To address this, we aim to build AI-assisted systems that simulate wheat pathologist evaluation and support remote diagnosis.
(2) Uncertain trust among wheat pathologists. Trust relationships are often incomplete or unknown, which can hinder consensus. We introduce a trust-aware model to handle missing trust values and enable reliable information propagation.
(3) Overlapping communities from diverse backgrounds. Wheat pathologists often belong to multiple communities due to differences in research focus or institutional roles. We propose an overlapping community detection method that better reflects real-world group structures.
(4) Fairness and adjustment cost in opinion updates. Excessive or unfair opinion adjustments reduce wheat pathologist engagement. We design an optimization approach that balances fairness utility and adjustment cost to enhance consensus quality and acceptance.

The main contributions of this paper are as follows:

(1) An AI-assisted wheat pathologist evaluation mechanism is constructed by uploading wheat condition maps from multiple regions to Claude 3.7. Role-playing and chain-of-thought reasoning are integrated to enhance diagnostic precision for wheat rust.
(2) A trust graph neural network (TGNN) is proposed to address incomplete trust relationships among wheat pathologists. The model incorporates multi-path trust propagation with learnable weights, improving the robustness of consensus formation.
(3) An overlapping community detection method is introduced to reflect the diverse backgrounds of wheat pathologists. The approach utilizes the Egret optimization algorithm to determine bounds and cluster numbers, combining three-cluster and K-means algorithms for accurate community identification.
(4) An optimization-based opinion adjustment framework is designed to balance fairness utility and adjustment cost across three types of uncertain robust group scenarios, promoting fair and cost-effective consensus outcomes.

Section 2 introduces the selected datasets, explaining their sources and relevance, and presents the data preprocessing approach based on simulating wheat pathology experts using AIGC. It also provides a detailed explanation of the proposed WCSE-AGC method. Section 3 describes the application of this method to the selected datasets, outlining key implementation steps and presenting the corresponding data. Section 4 discusses the broader applications of AI in agriculture, conducts a comparative analysis with existing methods to validate the advantages of the proposed approach, and includes a sensitivity analysis to evaluate its stability. The section concludes with a summary of findings and final remarks.

## 2. Data and Methods

This section provides an overall introduction to the data and the methods used in this study.

### 2.1. Data Acquisition and Preprocessing

This subsection presents the four publicly available datasets selected for the experiments and explains the rationale behind their selection. Subsequently, it provides a detailed description of the data preprocessing steps and the specific implementation of the evaluation matrix.

#### 2.1.1. The Overview of Data Sources

The four countries selected for this study, China, India, Ethiopia, and Turkey, demonstrate broad representativeness in terms of geographic scope and population distribution, spanning Asia, Africa, and Europe. In particular, China and India, as the two most populous countries in the world, significantly enhance the study’s practical coverage and scalability. The datasets from these countries collectively reflect diverse agricultural environments and disease pressures, lending the research outcomes greater generalizability across major wheat-producing regions worldwide.

The choice of the four datasets from China [13], India, Ethiopia, and Turkey is motivated by several important considerations, as summarized below:

- **Socio-agricultural context and disease prevalence:**
  - *China (Yangling, Shaanxi):* China’s complex terrain and diverse climates cause distinct wheat rust outbreaks, with cross-regional epidemics nearly every year causing about one million tons of yield loss annually [43]. Yangling, a key winter wheat region, experiences frequent leaf rust due to its climate. The dataset, annotated by experts, reflects real field conditions for effective disease monitoring.
  - *India (Indian Agricultural Research Institute (IARI), New Delhi):* Wheat is India’s second largest cereal after rice, with 31.4 million hectares planted and annual production around 107.6 million tons [44]. Northern India is a leaf rust hotspot. The dataset includes leaf rust and nitrogen deficiency images, supporting multi-task learning and capturing regional disease complexity.
  - *Ethiopia (Multiple Farms):* Wheat is a staple crop with growing production, over 7.2 million tons annually, 2.3 million hectares planted, and yields of 3.0 tons/hectare, ranking 18th worldwide [45]. The dataset captures multiple diseases under complex backgrounds, highlighting diagnosis challenges in resource-limited settings and aiding AI detection efforts.
  - *Turkey (Haymana, Ankara):* Wheat is a staple crop across almost all provinces, including spring and winter types, vital for food security and the economy [46]. The dataset, from a temperate continental climate zone, focuses on yellow rust with various severity levels, providing a robust benchmark for Eurasian wheat rust research.
- **Public data availability and device diversity:** Currently, publicly available wheat leaf rust image datasets specific to distinct geographic regions are limited, especially those collected using portable mobile devices. These four datasets collectively address this gap by providing high-quality images captured under diverse conditions and using various portable devices, enhancing the applicability of the study to real-world field scenarios.
- **Image content and standardization:** All datasets focus on individual wheat leaves to clearly display yellow rust symptoms, which minimizes background noise and environmental interference. This focused imaging approach facilitates accurate disease severity assessment. Furthermore, the adoption of a consistent image format across the datasets ensures a standardized basis for analysis, improving fairness and comparability despite regional differences in imaging conditions and field complexity.
- **Authoritative institutional sources:** Each dataset originates from reputable institutions with strong academic or research credentials, namely the Chinese Academy of Agricultural Sciences, the IARI, Ethiopian university collaborations, and the Turkish National Agricultural Research Institute. This ensures data credibility, scientific rigor, and high-quality annotations.

#### 2.1.2. Data Handling

Due to the limited availability of wheat pathologists specializing in wheat leaf rust, especially in large-scale agricultural production areas, it is not feasible to ensure wheat pathologist evaluation for every wheat-growing region. Monitoring and assessing wheat leaf rust typically require specialized knowledge and experience, but the existing number of wheat pathologists is insufficient to meet this demand, particularly in remote areas or developing countries. Furthermore, wheat pathologist evaluations are often constrained by individual experience, knowledge backgrounds, and adaptability to different environments, which can lead to inconsistencies and subjectivity in the assessment results. In this context, traditional methods relying on human evaluations are both costly and inefficient, especially when dealing with large-scale farm monitoring tasks. To address this issue, this study employs artificial intelligence, specifically the large language model Claude 3.7, to replace some wheat pathologist tasks, enabling efficient and accurate assessments without the need for a large number of wheat pathologists.

**Figure 9:**
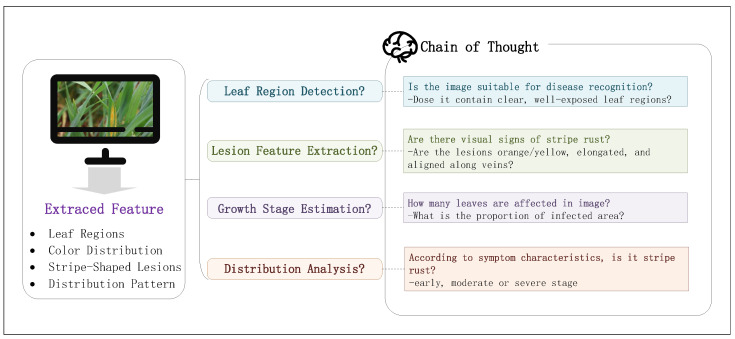
Chain-of-thought guidance for evaluating wheat stripe rust conditions.

The core of the chain-of-thought technique lies in guiding the model through incremental reasoning steps to enhance the transparency and accuracy of its decision-making process. By explicitly listing intermediate reasoning steps, the model can clearly demonstrate how it derives final conclusions from a series of observations, thus avoiding errors due to insufficient reasoning. This approach not only strengthens the model’s logical coherence but also improves the reliability of its generated results. As shown in Figure 9, the chain-of-thought structure allows the model to systematically organize the reasoning process, ensuring that the evaluation results align with expert-level standards.

**Figure 10:**
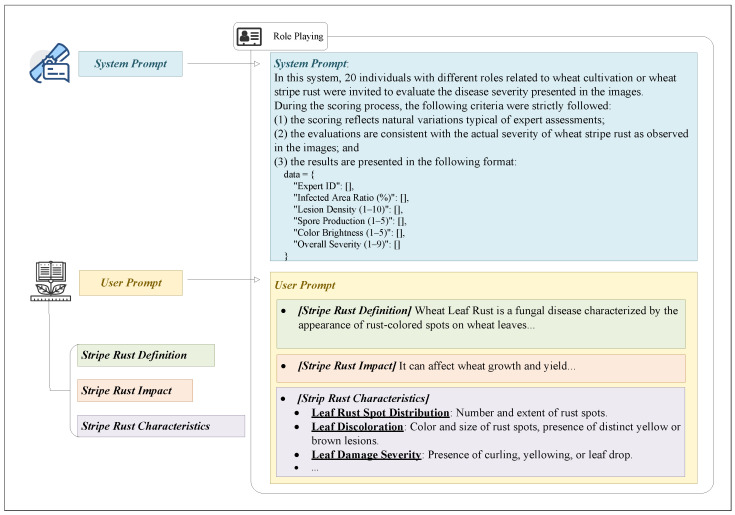
The framework of the role-playing-based wheat pathologist scoring system for wheat stripe rust.

The role-playing technique enables the model to simulate multiple expert roles related to wheat cultivation, mimicking the process of wheat pathologists’ assessment of wheat leaf rust. Each simulated role provides scores based on predetermined standards for evaluating the disease. This method models the multidimensional thinking of wheat pathologists and ensures that the evaluation results are not biased by a single wheat pathologist’s perspective. As illustrated in Figure 10, the role-playing technique allows the system to comprehensively score the evaluation criteria across different roles, thus providing objective and diverse assessment results.

To ensure the comprehensiveness and accuracy of the evaluation results, this study establishes five key assessment criteria, which are widely applicable in evaluating wheat leaf rust. The specific criteria are as follows:

(1) **Infection area ratio**: The percentage of leaf surface covered by rust lesions (0–100%).
(2) **Lesion density**: The number of lesions per square centimeter (rated on a scale of 1–10).
(3) **Spore production level**: The extent of spore production within lesions (rated on a scale of 1–5).
(4) **Lesion color vividness**: The brightness of yellow/orange lesions (rated on a scale of 1–5).
(5) **Overall severity**: A comprehensive severity score (rated on a scale of 1–9).

### 2.2. The Assessment of Wheat Rust Severity in Planting Areas Based on Wheat Pathologists’ Scoring and the CRP

This study proposes a consensus model designed to optimize wheat pathologists’ opinion adjustment within a social network framework, with a focus on the assessment of the severity of wheat stripe rust, a significant crop disease affecting wheat production. The model incorporates overlapping wheat pathologist communities and considers mutual trust and fairness in the opinion adjustment process, thereby enhancing both the reliability and objectivity of group decision-making.

**Figure 11:**
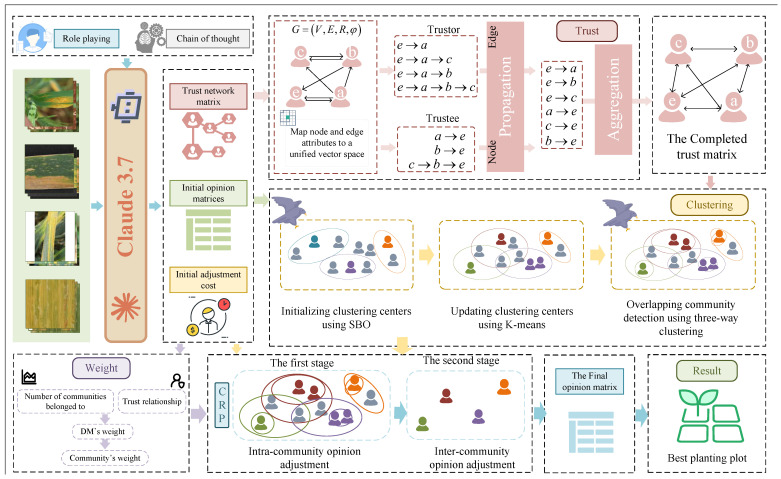
Overall framework of the planting plot selection method.

Wheat pathologist evaluations on the severity of wheat stripe rust were collected from four representative wheat-growing regions. These opinions were refined through a structured consensus mechanism, leading to an integrated severity ranking across regions. The overall evaluation framework, which leverages the collective insights of wheat pathologists, is illustrated in Figure 11.

#### 2.2.1. The Learnable and Composable Trust Propagation Based on GNNs

In wheat stripe rust assessment, the reliability of wheat pathologists’ opinions is a decisive factor. Trust relationships among them play a key role in influencing how their views are weighed during the consensus-reaching process. These relationships are often asymmetric and incomplete, making it essential to develop a robust method to complete the trust matrix. To address this, we propose a GNN-based framework that captures trust propagation and composability in a learnable manner. This framework maps node and edge attributes into a unified latent space, models trust propagation across trust chains (considering asymmetry), and aggregates trust information with attention mechanisms to complete the trust matrix and improve consensus reliability.

In this fully trusted network, denoted as G=(V,E,R,ϕ), nodes and edges have heterogeneous attributes, which may differ in dimensionality or reside in different vector spaces. To address this, attributes are transformed into a unified dimensional space before being fed into the GNN.

**Remark** **1** **(Node and edge attribute projection).** *A linear transformation is applied to all nodes to project their attributes into a unified space:*

(1) $$h_{v}=W_{node}\cdot x_{v},$$

*where $x_{v}$ is the attribute vector of v, $h_{v}$ is its transformed representation, and $W_{node}$ is the learnable matrix. For edge types, the transformation for the i-th edge is*

(2) $$r_{i}=W_{edge-i}\cdot x_{edge-i},$$

*where $x_{edge-i}$ is the attribute vector of the i-th edge type, and $W_{edge-i}$ is the corresponding learnable matrix. In cases of missing attributes, these are initialized as random vectors, which are treated as learnable parameters in the network.*

Trust relationships among pathologists are dynamically updated and propagated through existing trust chains in the social network. Within the GNN, trust chains are used for message passing with a propagation depth limited by a predefined threshold *K* to prevent degradation due to excessively long chains.

**Remark** **2** **(Forward trust propagation).** *For a receiver node v, suppose there exists a trust chain of length k, denoted as p:u→…→v. The trust value information from u to v needs to be updated based on the edges along this trust chain and the information from the initial node of the chain. The attributes of nodes and edges are processed in a manner similar to the RotatE method, where they are embedded in the complex plane.*

(3) $$h_{v}^{p}=h_{u}\circ r_{1}\circ \ldots \circ r_{k},$$

*where $h_{u}$ is the embedding of the initial node u, $r_{i}$ represents the edge embedding of the i-th edge, and ∘ denotes the Hadamard (element-wise) product.*

**Remark** **3** **(Reverse trust propagation).** *Let $h_{u},r_{1},\ldots ,r_{k}\in C^{d}$, where $C^{d}$ denotes the complex plane and $\left|r_{i}\right|=1$. The Hadamard product of the edge embeddings $r_{1}\ldots r_{k}$ can be viewed as a composite relation specific to the trust chain p. The embedding $h_{p}^{v}$ represents the information received by the node v along the chain p.*

*Trust is inherently asymmetric. Specifically, the node v also acts as a delegator in the trust graph. Therefore, comprehensive node embedding should incorporate both roles of being a trustee and a delegator. For the same trust chain p with the node v as the head (i.e., $v\overset{r_{1}}{\to }\ldots \overset{r_{k}}{\to }u$), TGNN computes*

(4) $$h_{p}^{v}=h_{u}\circ r_{k}^{*}\circ \ldots \circ r_{1}^{*}.$$

*The conjugate vector $r_{i}^{*}$ represents the inverse relation of $r_{i}$ (i.e., trust and trusted). This ensures that $h_{p}^{v}$ captures the information received by node v in its role as a trustor within the trust chain p.*

Multiple trust chains may exist between any two wheat pathologists. These chains can contribute unequally to the overall trust value due to diverse interaction patterns. To model this, a chain-type-level attention mechanism is applied that aggregates trust information across chains while differentiating their significance. The model further captures the trustee and delegator roles separately before combining them.

**Remark** **4** **(Trust aggregation).** *Let v be a node and $P_{j}$ the set of trust chains of type j. The aggregated embedding for type j is computed as*

(5) $$h_{P_{j}}^{v}=W_{P_{j}}\cdot p_{j}\left(h_{v}\right)+\sum_{p_{j}\in P_{j}}h_{p}^{v},$$

*where $p_{j}\left(\cdot \right)$ is a mapping function for chain type j, $W_{P_{j}}$ is a learnable transformation matrix, and $h_{p}^{v}$ is the embedding propagated along the trust chain $p_{j}$.*

*To evaluate the importance of each chain type, the following attention score is computed:*

(6) $$w_{P_{j}}=\frac{1}{\left|V\right|}\sum_{v\in V}q^{T}\cdot \tanh\left(W_{\mathrm{attn}}\cdot h_{P_{j}}^{v}+B\right),$$

*where $W_{\mathrm{attn}}$, B, and q are shared learnable parameters. These scores are normalized via the softmax function:*

(7) $$\alpha_{P_{j}}=\frac{\exp(w_{P_{j}})}{\sum_{c=1}^{k}\exp\left(w_{P_{c}}\right)}.$$

*The final node embedding from the trustee perspective is calculated as*

(8) $$Z=\sum_{j=1}^{k}\alpha_{P_{j}}\cdot H_{P_{j}},$$

*where $H_{P_{j}}$ denotes the embedding matrix for chain type j.*

*To account for the delegator perspective, an analogous aggregation is performed:*

(9) $$Z^{\prime }=\sum_{j=1}^{k}\alpha_{P_{j}}^{\prime }\cdot H_{P_{j}}^{\prime }.$$

*Here, $H_{P_{j}}^{\prime }$ represents embeddings from the delegator role, and $\alpha_{P_{j}}^{\prime }$ denotes the corresponding attention weights.*

#### 2.2.2. Weight Determination and CRP Under the Influence of Trust Relationships

This section analyzes trust dynamics within the wheat pathologist community, focusing on how overlapping community memberships and trust levels affect the weighting of their opinions in assessing wheat stripe rust severity.

Wheat pathologists often belong to specialized communities such as pest control, cultivation, or pathology. Trust within these communities significantly influences the impact of their opinions in decision-making.

For instance, a pathologist trusted for their expertise in disease management holds greater influence in rust control decisions. Those involved in multiple communities contribute broader insights, integrating knowledge across fields, which enhances consensus quality.

Trust relationships in overlapping communities further adjust opinion weights, reflecting trust levels in each community to better influence the final consensus.

Modeling these trust interactions ensures opinions are properly weighted, leading to more reliable consensus and targeted crop protection strategies.

Understanding the interplay of trust and community membership is crucial for optimizing consensus accuracy and delivering a comprehensive assessment of wheat stripe rust severity.

This section introduces a method for determining wheat pathologist weights in wheat research, focusing on the influence of trust relationships and involvement in overlapping pathologist communities. The trust received from peers and multi-community participation are key factors influencing their relative weights in discussions and decisions on pest control, planting technology, and variety improvement.

**Remark** **5** **(Individual weight of wheat pathologists).** *The computation of individual wheat pathologist weights incorporates two key factors: (1) the cumulative trust received from other wheat pathologists and (2) the degree of overlapping community participation. These are formulated as follows.*

***Trust-based influence:***

(10) $$WT_{i}=\sum_{j=0}^{N}T_{ji},\;i\neq j.$$

*This reflects the trust received by wheat pathologist i from all other wheat pathologists.*

***Overlap-based adjustment:***

(11) $$WO_{i}=1+\exp\left(\gamma \cdot overlap(i)\right),$$

*where overlap(i) represents the number of communities to which wheat pathologist i belongs, and γ is a tunable sensitivity parameter.*

***Combined intermediate weight:***

(12) $$W_{i}^{\prime }=\frac{WT_{i}+WO_{i}}{2}.$$

***Final normalized wheat pathologist weight:***

(13) $$W_{i}=\frac{W_{i}^{\prime }}{\sum_{j=0}^{N}W_{j}^{\prime }}.$$

***Community-level weight:*** *After obtaining the weights of individual wheat pathologists, the overall weight of a community is computed by summing the weights of all wheat pathologists within that community.*

Before determining the optimal wheat planting location, it is necessary to evaluate whether sufficient consensus has been achieved among regional wheat pathologists. Traditional soft consensus methods assess agreement based on opinion differences. However, wheat pathologists’ trust relationships also reflect the degree to which a pathologist accepts collective judgment. To this end, a modified consensus evaluation framework is proposed, which incorporates both opinion divergence and mutual trust to provide a more comprehensive consensus measurement.

The traditional soft consensus level only considers the distance between a pathologist’s revised opinion and the collective opinion. If the deviation is within an acceptable threshold (less than twice the collective opinion), a normalized consensus score is assigned; otherwise, the pathologist is considered completely inconsistent with the group. The collective opinion is calculated as a weighted average of all pathologists’ inputs.

However, this does not account for how much a pathologist trusts the group. A highly trusted pathologist, or one who has high trust in others, should influence and be influenced more in consensus building. Therefore, a trust-based modification is added. The individual trust level is calculated from the average trust a pathologist receives from others, while the overall trust level is the average trust across the group. The final individual consensus level is then adjusted by considering both opinion deviation and trust deviation. The group consensus level is the average of all individual consensus scores.

**Remark** **6** **(Group consensus level).** *The collective opinion $O^{c}$ is given by the weighted sum:*

(14) $$O^{c}=\sum_{i=0}^{M}W_{i}\cdot O_{i}.$$

*The individual trust level $T_{i}$ of wheat pathologist i is calculated as*

(15) $$T_{i}=\frac{\sum_{j=0,j\neq i}^{n}t_{ji}}{n-1}.$$

*The overall group trust level T is*

(16) $$T=\frac{\sum_{i=0}^{n}T_{i}}{n}.$$

*The trust-enhanced consensus level $CL_{i}$ is defined as*

(17) $$CL_{i}=\left\{\begin{array}{cc} \frac{2-\frac{|O^{c}-\bar{O_{i}}|}{O^{c}}-\frac{|T-T_{i}|}{T}}{2}, & \mathrm{if}\;\bar{O_{i}}<2O^{c}\wedge T_{i}<2T\\ 0, & \mathrm{otherwise} \end{array}\right..$$

*The overall group consensus level GCL is calculated by*

(18) $$GCL=\frac{\sum_{i=0}^{n}CL_{i}}{n}.$$

#### 2.2.3. Overlapping Community Detection Based on SBO K-Means and Three-Way Clustering

In wheat stripe rust assessment, wheat pathologists’ opinions may vary due to subjective biases and other influencing factors. To improve accuracy, incorporating multiple wheat pathologists’ opinions is essential. However, as the number of wheat pathologists increases, the decision-making process becomes more complex. To address this, clustering techniques can be employed to group wheat pathologists based on their opinions and trust levels, thus improving the efficiency of group consensus.

A common challenge in this process is the presence of overlapping communities, where a wheat pathologist belongs to multiple groups. This overlap is beneficial, as it reflects shared perspectives between groups and facilitates the adjustment of opinions across them. Such communities help reduce the cost of reconciling differing opinions, leading to a more accurate assessment of wheat stripe rust severity.

Traditional clustering methods, such as K-means and metaheuristic algorithms, often struggle with overlapping communities. However, by combining K-means with the SBO algorithm, cluster centers can be effectively identified, and a three-way clustering approach can be applied to partition wheat pathologists into core, boundary, and exclusion regions. This approach allows for the detection of overlapping communities, which is crucial for improving consensus in LSGDM, particularly in the context of wheat infection assessment.

The SBO algorithm is inspired by the hunting behavior of the secretary bird, a distinctive African raptor. It operates in three primary stages: initialization, hunting strategy, and escape strategy.

**Remark** **7** **(Initialization phase).** *Random initialization updates the position of each secretary bird in the search space:*

(19) $$X_{i,j}=lb_{j}+r\times \left(ub_{j}-lb_{j}\right),\;i=1,2,\ldots ,N,\;j=1,2,\ldots ,Dim,$$

*where ub and lb are the upper and lower bounds and r∈[0,1] is a random number.*

**Remark** **8** **(Hunting phase).** *The hunting strategy includes three intervals:*

*(1) **Prey searching*** *:*

(20) $$x_{i,j}^{new,P1}=x_{i,j}+\left(x_{random\_1}-x_{random\_2}\right)\times R_{1},\;t<\frac{T}{3}.$$

*(2) **Prey exhaustion*** *:*

(21) RB=rand(1,Dim)

(22) $$x_{i,j}^{new,P1}=e^{{\left(\frac{t}{T}\right)}^{4}}\times \left(RB-0.5\right)\times \left(x_{best}-x_{i,j}\right)+x_{best},\;\frac{T}{3}<t<\frac{2T}{3}.$$

*(3) **Prey attack*** *:*

(23) $$x_{i,j}^{new,P1}=x_{best}+{\left(1-\frac{t}{T}\right)}^{\frac{2t}{T}}\times x_{i,j}\times RL,\;t>\frac{2T}{3},$$

(24) RL=0.5×Levy(Dim).

**Remark** **9** **(Escape phase).** *The escape strategy involves camouflage or sudden running:*

(25) $$X_{i,j}^{new,P2}=\left\{\begin{array}{cc} x_{best}+\left(2\times RB-1\right)\times {\left(1-\frac{1}{T}\right)}^{2}\times x_{i,j}, & \mathrm{if}\;rand<r_{i}\\ x_{i,j}+R_{2}\times \left(x_{random}-K\times x_{i,j}\right), & \mathrm{otherwise} \end{array}\right.,$$

(26) K=round(1+rand(1,1)).

To handle uncertainty in clustering, three-way clustering is used. Unlike traditional methods that assign each data point to a single cluster, three-way clustering categorizes elements into three regions: core, boundary, and exclusion. This method enhances robustness in uncertain environments.

**Remark** **10** **(Three-way clustering).** *Let $U=\{x_{1},x_{2},\ldots ,x_{n}\}$ be the dataset. The result of three-way clustering is*

(27) $$T=\left\{\left(Co\left(c_{1}\right),Fr\left(c_{1}\right)\right),\left(Co\left(c_{2}\right),Fr\left(c_{2}\right)\right),\ldots ,\left(Co\left(c_{k}\right),Fr\left(c_{k}\right)\right)\right\}.$$

*Subject to*

*(1)*  *$Co(C_{i})\neq \emptyset$,*
*(2)*  *${⋃}_{i=1}^{k}\left(Co\left(C_{i}\right)\cup Fr\left(C_{i}\right)\right)=U$,*
*(3)*  *$Co\left(C_{i}\right)\cap Co\left(C_{j}\right)=\emptyset$, for i≠j.*

*If $Fr(C_{i})=\emptyset$, it degenerates into two-way clustering.*

The K-means algorithm is widely used to partition data into *k* clusters by minimizing the sum of squared distances within clusters. The basic steps include

**Remark** **11** **(K-means clustering).**

*1.*  *Randomly initialize k cluster centers.*
*2.*  *Assign each data point to the nearest cluster using Euclidean distance:*
(28) $$d=\sqrt{\sum_{i=1}^{m}{\left(x_{i}-y_{i}\right)}^{2}}.$$
*3.*  *Recalculate centers and iterate until convergence.*

To overcome the local optima limitation of K-means, the SBO algorithm is used to optimize initial cluster centers. With *k* communities, set ub=k and lb=1, generating an N×N matrix with random integers representing community assignments.

**Remark** **12** **(Initial clustering).**

(29) $$C=\left[\begin{array}{cccc} c_{1,1} & c_{1,2} & \ldots & c_{1,n}\\ c_{2,1} & c_{2,2} & \ldots & c_{2,n}\\ \vdots & \vdots & \ddots & \vdots \\ c_{N,1} & c_{N,2} & \ldots & c_{N,n} \end{array}\right].$$

*This matrix serves as the initialization for K-means and three-way clustering, enabling the identification of overlapping communities among wheat pathologists.*

In standard K-means, Euclidean distance is commonly used. However, for high-dimensional opinion data with many criteria, we use an improved Manhattan distance to simplify and stabilize the computation. Let $X=[x_{1},...,x_{N}]$ and $Y=[y_{1},...,y_{N}]$ represent the opinions of two wheat pathologists. The distance between them is calculated as

**Remark** **13** **(Clustering center identification).**

(30) $$d_{x,y}=\frac{1}{N}\sum_{i=1}^{N}\left|x_{i}-y_{i}\right|.$$

*Let M be the number of wheat pathologists in a sub-community. Compute the distance matrix:*

(31) $$D={\left[d_{i,j}\right]}_{M\times M}.$$

*Then, using the trust matrix $T=[t_{ji}]$, calculate the comprehensive distance from each wheat pathologist i to others as*

(32) $$DT_{i}=\frac{1}{M}\sum_{j=1}^{M}d_{ij}\left(1-t_{ji}\right).$$

*The wheat pathologist with the smallest $DT_{i}$ is chosen as the cluster center. Iterate the process until centers stabilize.*

To identify overlapping areas between core and boundary regions, upper and lower bounds (UB, LB) are initialized using the principles in Remark 10. These bounds are adaptively refined based on the consensus level of the group.

The SBO’s hunting and escape strategies (see Remarks 8 and 9) are then employed to iteratively adjust these bounds. Parameters are updated as in the natural prey capture process of the secretary bird, and any improvements in partition quality are preserved.

Finally, based on the optimized bounds, the community partitioning is finalized, accurately reflecting overlapping community structures and enhancing the overall consensus process.

#### 2.2.4. Fairness-Conscious Optimization for Robust Group Decision-Making

In the context of wheat stripe rust assessment, wheat pathologists are often required to adjust their opinions when confronted with new information or differing viewpoints. Given that the severity of wheat stripe rust is influenced by a variety of environmental and biological factors, reaching group consensus is often complex and uncertain. In such situations, fairness perception and adjustment costs become critical factors guiding the decision-making process.

Fairness is particularly important in wheat pathologists’ assessments of wheat stripe rust, as wheat pathologists are more likely to accept and cooperate with opinion adjustments when they perceive the process as fair. In this context, fairness can be reflected in ensuring that each wheat pathologist’s opinion is appropriately weighted, preventing any single individual’s view from dominating unfairly and maintaining transparency and equity across different regions. For example, if a wheat pathologist feels that their experience in diagnosing wheat diseases is undervalued in the decision-making process, they may be reluctant to cooperate or accept the final consensus. This could compromise the accuracy of the rust severity assessment and hinder the effectiveness of subsequent disease control measures.

At the same time, each opinion adjustment comes with certain costs, which vary depending on the specific situation. These costs may arise from the need for additional resources to collect field data or the time required for wheat pathologists to reassess their opinions in light of new information. For instance, in assessing wheat stripe rust, a wheat pathologist may encounter difficulties due to the lack of detailed data on disease progression or infection density. If certain planting regions lack real-time imagery of infected wheat, the wheat pathologist may be forced to rely on less accurate data or invest additional time and resources to obtain more reliable information. This uncertainty introduces potential adjustment costs that can delay or complicate consensus formation.

In light of these challenges, it is essential to incorporate fairness considerations and control adjustment costs effectively during the evaluation of wheat stripe rust severity. Specifically, a robust adjustment mechanism should be established to account for the costs of acquiring new information, addressing disagreements among wheat pathologists, and ensuring the fair representation of each wheat pathologist’s viewpoint. This not only minimizes the psychological and resource burden on individuals but also enables the final consensus to more accurately reflect the collective expertise of the group. As a result, the evaluation of rust spread and severity becomes more precise and reliable, providing a solid basis for disease control strategies and resource allocation.

Therefore, in such contexts, an optimized adjustment strategy that integrates fairness and cost control is essential for achieving efficient and rational decision-making. By reducing informational and psychological barriers and fostering collaboration among wheat pathologists, a more accurate, comprehensive, and actionable consensus on wheat stripe rust can be achieved.

**Remark** **14** **(Fairness).** *The Procedural Fairness and Social Preference Model emphasizes fairness in income distribution. It is characterized by a small number of parameters, a simple structure, and strong predictive capabilities. Within this model, individuals evaluate fairness by comparing their own outcomes with those of others. When a wheat pathologist’s income is lower than that of others, feelings of envy may arise; conversely, when their income is higher, emotions such as pride or sympathy may be experienced. The model’s computational equation is presented as follows:*

(33) $$U_{i}=x_{i}-\frac{\alpha_{i}}{n-1}\sum_{j\neq i}^{n}\max\left(x_{j}-x_{i},0\right)-\frac{\beta_{i}}{n-1}\sum_{j\neq i}^{n}\max\left(x_{i}-j,0\right).$$

*In the above equation, $U_{i}$ denotes the utility of individual i, and $x_{i}$ represents the income of individual i. The envy aversion coefficient $\alpha_{i}\in \left[0,1\right]$, and the sympathy/pride preference coefficient $\beta_{i}\in \left[-1,1\right]$. Typically, these two parameters satisfy the condition $\alpha_{i}>\beta_{i}$.*

*When the two parameters take different values, they reflect different behavioral states of the wheat pathologist.*

- *When $\beta_{i}\in \left[-1,0\right]$, the fairness utility of the wheat pathologist consists of compensation, the negative utility caused by envy preferences, and the positive utility generated by the pride coefficient.*
- *When $\beta_{i}\in \left[0,1\right]$, the fairness utility consists of compensation, the negative utility caused by envy preferences, and the negative utility resulting from the sympathy coefficient.*

The uncertainty of the unit adjustment cost $c_{i}$ for wheat pathologist $d_{i}$ is determined using an uncertain set *U*, represented as

**Remark** **15** **(Adjustment cost).** *The uncertain set U is given by*

(34) $$U=\left\{c_{i}=c_{i}^{0}+\xi_{i}c_{i}^{l}:\xi_{i}\in U,i\in N\right\}.$$

*The fairness utility for wheat pathologist $d_{i}$ is*

(35) $$A=\frac{\alpha_{i}\sum_{j\neq i}^{n}\max\left(f_{j}\left(o_{j},\bar{o_{j}}\right)-f_{i}\left(o_{i},\bar{o_{i}}\right),0\right)}{n-1}.$$

(36) $$B=\frac{\beta_{i}\sum_{j\neq i}^{n}\max\left(f_{i}\left(o_{i},\bar{o_{i}}\right)-f_{j}\left(o_{j},\bar{o_{j}}\right),0\right)}{n-1}.$$

*The final fairness utility is*

(37) $$F^{\left(\alpha_{i},\beta_{i}\right)}\left(\bar{o_{i}},c_{i}\right)=f_{i}\left(o_{i},\bar{o_{i}}\right)-A-B.$$

*The fairness decision is*

(38) $$FD^{\left(\alpha_{i},\beta_{i}\right)}\left(\bar{o_{i}},c_{i}\right)=\left\{\begin{array}{c} 0,\;\mathrm{if}\;F^{\left(\alpha_{i},\beta_{i}\right)}\left(\bar{o_{i}},c_{i}\right)<0\\ \frac{F^{\left(\alpha_{i},\beta_{i}\right)}\left(\bar{o_{i}},c_{i}\right)}{f_{i}\left(o_{i},\bar{o_{i}}\right)},\;\mathrm{if}\;0<F^{\left(\alpha_{i},\beta_{i}\right)}\left(\bar{o_{i}},c_{i}\right)<f_{i}\left(o_{i},\bar{o_{i}}\right)\\ 1,\;\mathrm{if}\;f_{i}\left(o_{i},\bar{o_{i}}\right)\leq F^{\left(\alpha_{i},\beta_{i}\right)}\left(\bar{o_{i}},c_{i}\right) \end{array}\right..$$

**Remark** **16** **(The robust group).** ***Box Uncertainty Set:***

(39) $$U^{Box}=\left\{{\xi :\|\xi \|}_{\infty }\leq \tau ,\tau >0\right\}.$$

*The corresponding optimization equation considering fairness effects is*

(40) $$\begin{array}{cc} \; & \max\mu \\ \; & s.t.\\ \; & \sum_{i=1}^{n}c_{i}^{0}|o_{i}-\bar{o_{i}}|+\tau \sum_{i=1}^{n}|c_{i}^{l}\left|\right|o_{i}-\bar{o_{i}}|\leq B,\\ \; & 1-\frac{|o^{c}-\bar{o_{i}}|}{o^{c}}\geq \theta ,\\ \; & o^{c}=\sum_{i=1}^{n}w_{i}^{1-\rho_{i}}\bar{o_{i}},\\ \; & a+\frac{\tau \sum_{j=1}^{n}|c_{j}^{l}\left|\right|o_{j}-\bar{o_{j}}|}{c_{i}^{0}\left|o_{i}-\bar{o_{i}}\right|}\leq \left(n-1\right)\left(1-\mu \right) \end{array}.$$

***Elliptical uncertainty set:***

(41) $$U^{Ellipsoid}=\left\{{\xi :\|\xi \|}_{2}\leq \Omega ,\Omega >0\right\}.$$

*The corresponding optimization equation considering fairness effects is*

(42) $$\begin{array}{cc} \; & \max\mu \\ \; & s.t.\\ \; & \sum_{i=1}^{n}c_{i}^{0}|o_{i}-\bar{o_{i}}{|+\Omega ∥\omega ∥}_{2}\leq B,\\ \; & 1-\frac{|o^{c}-\bar{o_{i}}|}{o^{c}}\geq \theta ,\\ \; & o^{c}=\sum_{i=1}^{n}w_{i}^{1-\rho_{i}}\bar{o_{i}},\\ \; & a+\frac{{\Omega ∥\omega ∥}_{2}}{c_{i}^{0}\left|o_{i}-\bar{o_{i}}\right|}\leq \left(n-1\right)\left(1-\mu \right) \end{array}.$$

***Polyhedral uncertainty set:***

(43) $$U^{polyhedron}=\left\{{\xi :\|\xi \|}_{1}\leq \Gamma ,\Gamma >0\right\}.$$

*The corresponding optimization equation considering fairness effects is*

(44) $$\begin{array}{cc} \; & \max\mu \\ \; & s.t.\\ \; & \sum_{i=1}^{n}c_{i}^{0}\left|o_{i}-\bar{o_{i}}\right|+z\Gamma +\sum_{l\in L}q_{l}\leq B,\\ \; & z+q_{l}\geq \sum_{i=1}^{n}|c_{i}^{l}\left|\right|o_{i}-\bar{o_{i}}|,\\ \; & 1-\frac{|o^{c}-\bar{o_{i}}|}{o^{c}}\geq \theta ,\\ \; & o^{c}=\sum_{i=1}^{n}w_{i}^{1-\rho_{i}}\bar{o_{i}},\\ \; & a+\frac{z\Gamma +\sum_{l\in L}q_{l}}{c_{i}^{0}\left|o_{i}-\bar{o_{i}}\right|}\leq \left(n-1\right)\left(1-\mu \right) \end{array}.$$

Detecting overlapping communities during community partitioning is crucial for wheat pathologists. By utilizing the wheat pathologists in the overlapping regions, the overall gap between the opinions of different communities can be effectively reduced, thereby decreasing the need for opinion adjustments between communities.

In this paper, the weight of overlapping individuals is increased to make the group opinion lean toward the overlapping individuals, which in turn influences the adjustment direction of other opinions, ultimately guiding the opinions of other individuals toward the overlapping individuals.

The opinion adjustment process in this paper is divided into two stages. In the first stage, opinion adjustments are made within each community, and the consolidated opinion within each group is provided as the initial opinion for the second stage.

In the second stage, the consolidated opinions of different communities are adjusted, and the final adjusted opinion is provided.

**Figure 12:**
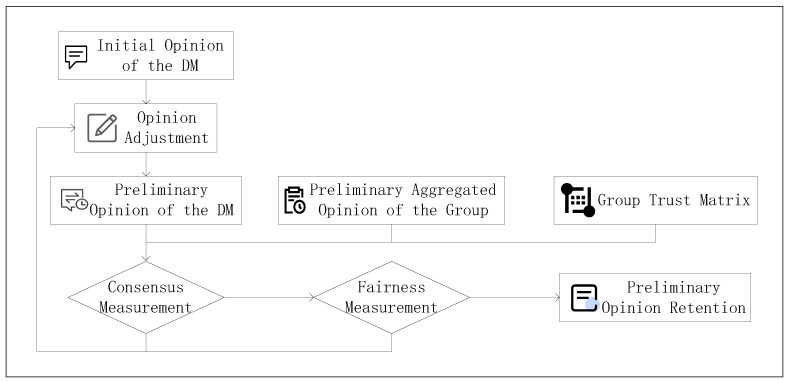
Implementation of the optimized adjustment process.

The specific methods for optimization and adjustment at each stage are shown in Figure 12.

## 3. Results

This section processes the specific datasets and presents the evaluation procedure for wheat leaf rust conditions at different locations.

### 3.1. Data Handling for Wheat Stripe Rust Image Sets

Due to the limited availability of wheat pathologists specializing in wheat leaf rust, it is not feasible to ensure wheat pathologist evaluation in every wheat-growing region in real-world scenarios. To address this issue, this study employs a large language model, utilizing chain-of-thought prompting and role-playing techniques to guide the model toward more accurate data interpretation. Given Claude 3.7’s advanced capabilities in processing images and multimodal data, it is selected in this work to perform the evaluation tasks.

In this study, four different locations were selected as candidate sites for decision-making based on publicly available wheat image datasets. These locations, hereafter referred to as $L=\left\{l_{1},l_{2},l_{3},l_{4}\right\}$, include an experimental field managed by the Holeta wheat farm located in Ethiopia; a wheat disease image dataset collected by the IARI in India; the Central Research Institute for Field Crops under the Ministry of Agriculture and Forestry of the Republic of Turkey, located near Haymana–Ankara, Turkey; and a wheat disease dataset collected by Northwest A&F University in Shaanxi, China.

The datasets selected from China, India, Turkey, and Ethiopia offer comprehensive coverage of wheat leaf rust under diverse agricultural, climatic, and socioeconomic conditions. These locations represent key wheat-producing regions with distinct leaf rust prevalence patterns, ensuring both geographical diversity and representativeness. All datasets were collected by authoritative institutions using field-based imaging methods, enhancing credibility and real-world applicability. Moreover, the images are standardized in format, focused on individual wheat leaves, and categorized by expert annotation, enabling the reliable training and evaluation of AI-based detection models. Collectively, these datasets provide a high-quality, balanced, and generalizable foundation for robust wheat leaf rust recognition research across different environmental and regional contexts.

In this study, 100 photos of wheat stripe rust were randomly selected from the four publicly available datasets mentioned above. These photos were sent to the large model simulating a wheat pathologist group, which provided a stripe rust score matrix for each photo. The average score for each wheat pathologist was then calculated based on the corresponding location and wheat pathologist score matrices. The average score for each wheat pathologist on the wheat stripe rust condition at a specific location is presented, and the processed results are shown in Appendix A.1.

### 3.2. Trust Matrix Completion and Wheat Pathlogist Adjustment Cost

The role-specific opinion adjustment costs were generated by simulating different roles using Claude 3.7, and the initial incomplete trust matrix was constructed based on the social network among these roles. The role of opinion adjustment costs is presented in the Table 1.

The initial incomplete trust matrix and the completed matrix are presented in Table 2. Each cell in the matrix represents the level of trust between two wheat pathologists. Values closer to dark red indicate a higher degree of trust, while those approaching dark blue indicate lower trust. In the initial trust matrix, cells marked as NaN denote unknown trust values between corresponding wheat pathologists, which are the targets for completion. The final trust matrix displays the fully estimated trust values among all wheat pathologists, obtained through the TGNN-based trust completion process.

### 3.3. Wheat Pathologist Clustering and Weight Calculation

**Figure 13:**
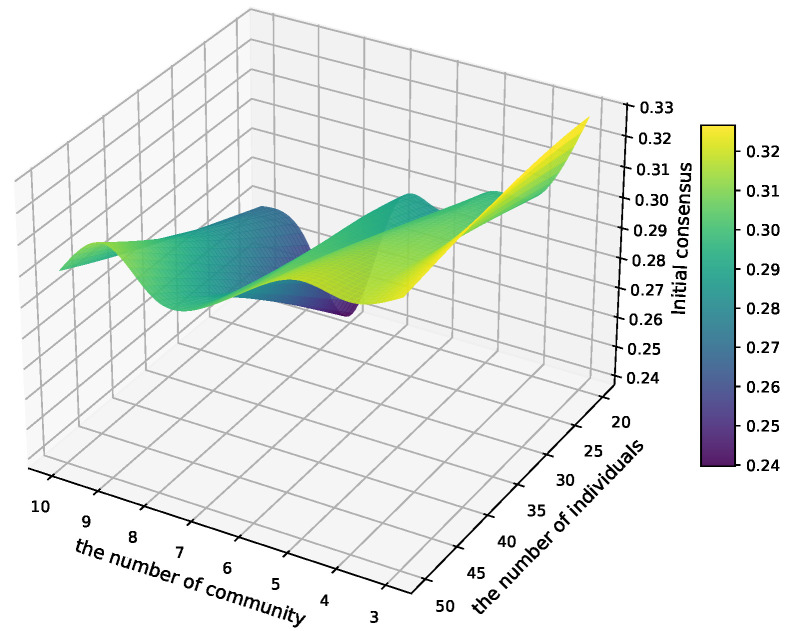
Initial consensus of clustering results for varying numbers of wheat pathologists and community divisions.

In order to identify the optimal value of *k*, the distribution of consensus degrees for various clustering outcomes is computed with respect to different numbers of wheat pathologists and cluster divisions, as illustrated in Figure 13. The x-axis indicates the number of communities in the clustering division, the y-axis denotes the number of wheat pathologists, and the z-axis represents the initial consensus levels of the clustering results.

To further evaluate the clustering quality under specific conditions, Table 3 presents the detailed initial consensus degrees corresponding to the case where the number of wheat pathologists is fixed at 20 (i.e., the y-axis value in Figure 13). The values in the table reflect the consensus outcomes across varying numbers of community divisions. A higher value indicates a greater level of initial consensus among the clustering results, implying a more coherent community structure.

This comparison helps in identifying the optimal number of communities for achieving the highest consensus when engaging a fixed number of experts. From the table, it can be observed that the clustering result with three communities achieves the highest consensus, suggesting it may represent the most internally consistent structure under this configuration.

As shown in Figure 13, the initial consensus level is highest when the clustering results are divided into three categories, specifically when the number of wheat pathologists is 20.

Table 4 presents the final clustering results derived from both the completed and the initial trust matrices. The left column displays the names of the identified communities, while the right column lists the wheat pathologists assigned to each community.

**Figure 14:**
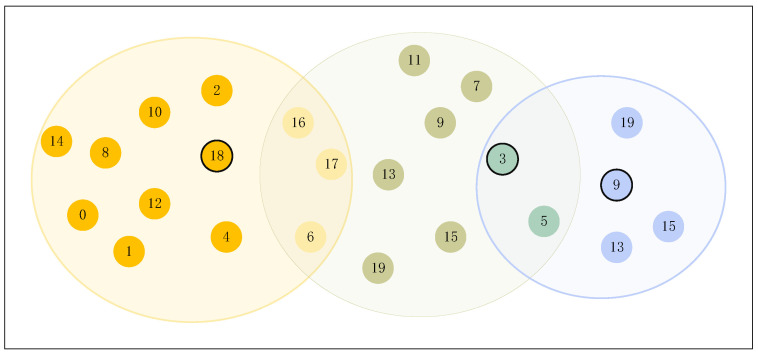
Visualization of clustering results.

In the clustering results, a subset of individuals belongs to overlapping communities, as illustrated in Figure 14. Individuals located within two or more colored circles are considered part of the overlapping regions, and the central representatives of each cluster are highlighted using bold black markers.

From Figure 14, it can be observed that the cluster centers for community1, community2, and community3 are $e_{9}$, $e_{3}$, and $e_{18}$, respectively. The overlapping wheat pathologists between communities3 and community2 include $e_{16}$, $e_{17}$, and $e_{6}$, whereas $e_{3}$ and $e_{5}$ are shared by communities2 and community1.

The weights of the wheat pathologists are determined based on the degree of overlap in the clustering results and the trust relationships among them. The calculated results are presented in Table 5.

The community weights are computed using the individual weights obtained from Table 5, and the results are presented in Table 6.

### 3.4. Adjustment of Opinions and Wheat Planting Suitability Ranking

**Figure 15:**
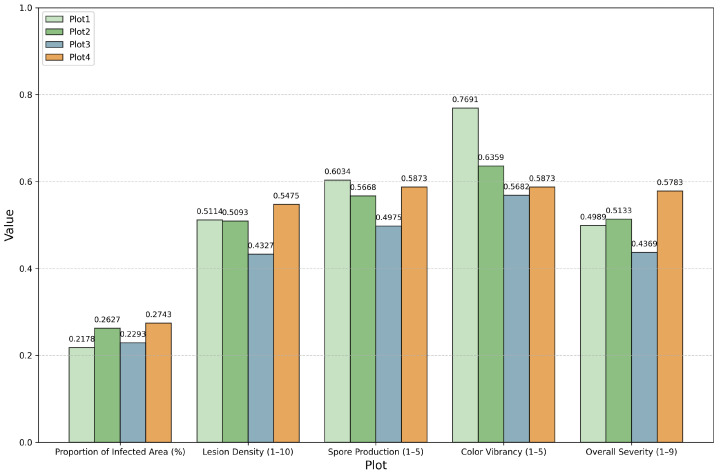
The scoring of final consensus Opinions.

In the first round of opinion adjustment, the opinions of wheat pathologists within the same community are revised until a consensus is achieved. The aggregated opinions within each community are then presented, as shown in Appendix B. Following this, a second round of opinion adjustment is applied to the aggregated opinions from different communities, and the final opinions regarding the four distinct planting locations are displayed in Figure 15.

As shown in Figure 15, the x-axis denotes various reference indicators, and different colors are used to represent the values for each location. The final scores for the results are indicated above the respective bars.

**Figure 16:**
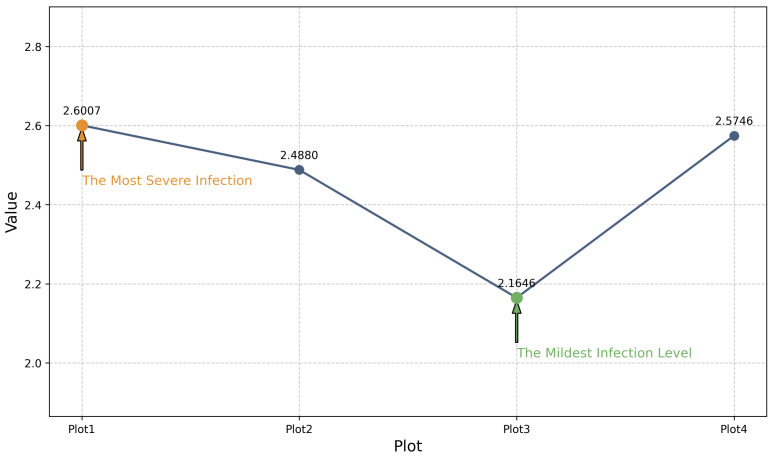
The final comprehensive score ranking.

The overall scores for wheat stripe rust across different locations are combined and presented for comparison in Figure 16, represented as a line chart. In this figure, higher scores correspond to more severe levels of stripe rust in the respective locations.

As shown in Figure 16, Plot1 experiences the most severe wheat stripe rust, whereas Plot4 exhibits the mildest symptoms. This finding can serve as a criterion for selecting planting locations, with the loss associated with planting in Plot4 being lower than in Plot1 under identical conditions.

## 4. Discussion

This section begins by discussing the practical applications of AI in agriculture, emphasizing its role in transforming crop disease identification and management. The strengths and limitations of using AIGC to simulate wheat pathologists are then explored. A comparative sensitivity analysis is conducted to evaluate the performance and stability of the proposed method in relation to existing approaches. Finally, conclusions are drawn.

### 4.1. Discussion of Experimental Results

**Figure 17:**
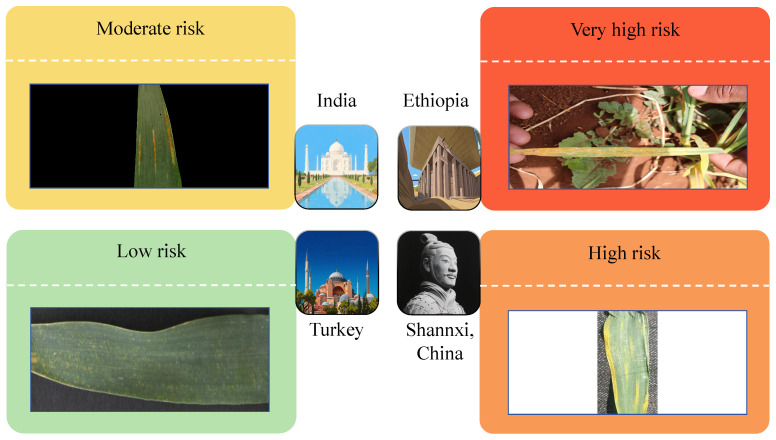
The final comprehensive score ranking.

Based on the final ranking results of stripe rust severity presented in the previous section, the relative levels of infection are illustrated in Figure 17.

As shown in the figure, Ethiopia exhibits the highest severity of stripe rust, indicating the need for increased attention to disease management during the cultivation process. The severity ranking, from most to least affected, is as follows: Ethiopia, China, India, and Turkey.

This ranking reflects not only the environmental and climatic conditions favorable to the spread of stripe rust in these regions but also highlights potential gaps in current disease monitoring and prevention strategies. For example, Ethiopia’s high severity may be attributed to limited access to resistant wheat varieties or insufficient early warning systems. In contrast, Turkey’s relatively mild severity suggests the presence of more effective disease control practices or environmental conditions less conducive to rust development. These results underscore the importance of tailoring disease management strategies to local contexts and call for increased international collaboration in sharing resistant germplasm, monitoring technologies, and field management practices to mitigate the global impact of stripe rust.

### 4.2. The Application of AI Technologies in Crop Disease Detection

Early pest and disease detection in agriculture has traditionally relied on manual inspection, where experienced practitioners assess crop health based on observable symptoms. While this method embodies rich empirical knowledge, it suffers from low efficiency, high subjectivity, and poor scalability—especially in large-scale farming scenarios. Variations in individual judgment further hinder the standardization and timeliness of disease management decisions.

To overcome these limitations, technology-driven approaches have emerged, including image recognition, sensor-based monitoring, AI/machine learning (ML), and remote sensing. Table 7 provides a comparative summary of these techniques based on their principles, strengths, weaknesses, and application contexts.

To further clarify practical performance differences, Table 8 evaluates each method across five key attributes: cost, ease of deployment, real-time monitoring, accuracy, and weather dependency. This allows agricultural stakeholders to make more informed decisions when selecting suitable detection tools.

Among these methods, AI-based approaches—especially deep learning—have shown transformative potential. Traditional ML techniques, such as support vector machines (SVMs), have been used for disease classification, with models like that of Prince et al. [54], combining CNN and SVM for explainable and efficient disease recognition. However, these methods often depend on handcrafted features and struggle with generalization in dynamic environments.

Deep learning models, particularly CNNs, automate feature extraction and deliver high classification accuracy. Vijayan et al. [55] proposed a hybrid CNN model for rice disease prediction, achieving notable accuracy gains. Similarly, Karim et al. [56] employed a lightweight CNN with Grad-CAM to enable the interpretable, real-time diagnosis of grape leaf diseases on edge devices. Despite their advantages, deep models are data-intensive, computationally demanding, and often opaque, making practical field deployment difficult without domain expertise and infrastructure.

Beyond CNNs, reinforcement learning (RL) is gaining traction for intelligent disease management. RL enables agents to adapt strategies based on environmental feedback. For instance, Chen et al. [57] combined RL with spectral imaging and SVM for soybean pest detection. Yet RL faces challenges such as high data requirements, slow learning convergence, and poor adaptability to fast-evolving disease conditions—especially in complex, resource-constrained agricultural settings.

In conclusion, while AI significantly enhances the scalability, accuracy, and automation of crop disease detection, each method presents trade-offs in terms of cost, complexity, interpretability, and adaptability. Selecting appropriate solutions requires balancing technological capabilities with real-world constraints and agricultural contexts.

### 4.3. Validation of AIGC Effectiveness and Discussion of Limitations

This section discusses the advantages and disadvantages of using AIGC to simulate wheat pathologists.

#### 4.3.1. Consistency Validation Between AIGC Outputs and Wheat Pathology Expert Ratings

To validate the effectiveness of Claude 3.7 in simulating disease severity assessments, we employed the publicly available Turkish dataset as a reference. This dataset contains numerous wheat leaf images annotated by experienced plant pathologists, providing a high-confidence “expert gold standard” for comparison with model-generated outputs.

A total of 1000 images were randomly selected from the dataset. Using role-playing prompts to simulate expert behavior, Claude 3.7 generated disease severity scores through chain-of-thought reasoning. For each image, the model produced a severity rating along with a corresponding reasoning process. These results were then compared against the original expert annotations to evaluate consistency.

The analysis showed that the Cohen’s Kappa coefficient between Claude 3.7 and the expert labels was 0.81, indicating substantial to almost perfect agreement. The overall classification accuracy reached 86.9%, further confirming the reliability of the model’s scoring performance.

These findings suggest that, when appropriately guided, Claude 3.7 is capable of effectively emulating the diagnostic reasoning patterns of plant pathology experts, supporting its potential for practical use in wheat disease assessment.

#### 4.3.2. Advantages and Limitations of AIGC-Generated Scoring Compared to Wheat Pathologists

Claude 3.7 and other AIGC models demonstrate significant advantages in assessing the severity of wheat leaf rust. Firstly, the models can rapidly process large volumes of images, greatly improving disease monitoring efficiency. Secondly, by adopting a unified evaluation standard, the influence of subjective human factors is reduced, thereby enhancing the consistency and reproducibility of the scoring results. Additionally, through role-playing and chain-of-thought prompting, the models are able to simulate expert reasoning processes, increasing the transparency and interpretability of the scoring, which is beneficial for assisting disease diagnosis and related training.

However, AIGC models also have certain limitations. Due to the lack of accumulated domain experience and intuition possessed by human experts, the models show reduced accuracy when dealing with images that have ambiguous boundaries or complex backgrounds. The design of the prompts greatly affects model performance, and slightly improper guidance may lead to scoring bias. In real agricultural environments, challenges such as leaf occlusion and multiple co-occurring diseases still test the model’s robustness. Moreover, the limited availability of high-quality expert-annotated data restricts comprehensive validation of the model’s capabilities. Therefore, while AIGC shows promising potential in assisting disease assessment, it is more suitable as a complementary tool to experts rather than a complete replacement. Future work may consider combining AI and expert review in a hybrid diagnostic approach to enhance overall effectiveness.

### 4.4. Comparative Analysis

In this subsection, each component of the proposed method is either replaced or removed to evaluate its contribution, with the analysis divided into two parts: clustering and consensus adjustment.

To validate the effectiveness of the overlapping sub-community detection approach described in this paper, the original method is denoted as m0. When the clustering method in m0 is replaced with the SBO algorithm, the variant is referred to as m1; replacing it with K-means clustering results in m2; and using three-way clustering instead yields m3. Furthermore, combining the SBO algorithm with K-means clustering is denoted as m4; combining SBO with three-way clustering is denoted as m5; and combining K-means with three-way clustering results in m6. In addition, replacing the SBO algorithm with a genetic algorithm results in m7, and replacing it with the hippo optimization algorithm yields m8. The evaluation results of the clustering performance under these different settings are presented in Table 9.

As shown in the table above, the clustering method proposed in this paper outperforms methods m1, m2, m3, and m4 by enabling the detection of overlapping sub-communities, and it also achieves a higher average level of community consensus. Compared to method m5, although both approaches support overlapping sub-community detection, the proposed method demonstrates superior efficiency and a higher level of consensus within communities. In contrast to method m6, although the clustering efficiency is roughly comparable, the proposed method still achieves a higher average community consensus, which can further enhance the efficiency of consensus adjustment. Similarly, compared to method m7, the proposed method shows better performance in both accuracy and stability, since standard genetic algorithms have limited capabilities in exploring the solution space, often resulting in suboptimal clustering outcomes. As for method m8, although the hippo optimization algorithm provides more comprehensive exploration and yields relatively competitive clustering results, its optimization process involves more complex formulations, leading to increased computational time. In contrast, the proposed method strikes a better balance between accuracy and efficiency, making it more suitable for practical large-scale decision-making scenarios.

In order to further verify the performance of the proposed method relative to existing approaches, a comparative analysis was carried out, with the corresponding results shown in Table 10.

Table 10 demonstrates that the proposed method incorporates a broader set of indicators during the opinion adjustment process and explicitly accounts for LSGDM scenarios. Despite requiring fewer adjustment rounds, it consistently achieves a higher final consensus level.

### 4.5. Sensitivity Analysis

This subsection investigates the stability of the proposed method by performing multiplicative adjustments to the parameter values and analyzing their effects on the final outcomes.

Table 11 presents the results of the sensitivity analysis conducted on the degree of missing trust values. Different levels of missing data (30%, 50%, and 70%) were simulated to examine the robustness of the final plot ranking.

As shown in the table, the ranking results remain consistent across all scenarios, indicating that the proposed decision-making framework is resilient to incomplete trust information.

Table 12 presents the results of the sensitivity analysis conducted on parameter γ, which controls the weight adjustment in the aggregation process. The analysis tests a range of values (0.5, 1, 2, and 3) to examine the impact of γ on the final ranking of the candidate plots.

As shown in the table, the final ranking remains unchanged across different values, indicating that the proposed method is robust with respect to variations in this parameter.

Table 13 presents the results of the sensitivity analysis conducted on parameter *k*, which determines the number of clusters in the K-means algorithm used during the decision-making process. The values of *k* were varied from 3 to 6 to evaluate the impact of clustering granularity on the final plot ranking.

As shown in the table, the ranking remains stable across different values of *k*, demonstrating the robustness of the proposed method against changes in the clustering parameter.

Table 14 presents the results of the sensitivity analysis conducted on parameter UB, which represents the upper bound in the three-way decision thresholding mechanism. The lower bound LB was kept constant at 0.43, while the upper bound UB was varied from 0.48 to 1.92 to evaluate its influence on the final ranking outcome.

As illustrated in the table, the final ranking remains unchanged despite changes in UB, suggesting that the decision-making framework maintains strong robustness with respect to the threshold settings.

Table 15 presents the results of the sensitivity analysis conducted on parameter LB, the lower bound in the three-way decision thresholding process. While the upper bound UB was fixed at 0.96, the value of LB was varied from 0.21 to 0.86 to assess its impact on the final ranking.

As shown in the table, the ranking of plots remains consistent across all tested values, indicating that the proposed method is robust to variations in the lower threshold setting.

### 4.6. Conclusions

This study presents a suite of methods that demonstrate substantial theoretical and practical contributions to the decision-making process for wheat stripe rust management, offering multifaceted advancements to the agricultural sector. The developed AI-assisted wheat pathologist evaluation mechanism leverages multi-regional wheat imagery uploaded to Claude 3.7 and integrates role-playing with chain-of-thought reasoning, effectively overcoming the limitations of traditional manual diagnosis characterized by inefficiency and subjectivity. This innovation enables the rapid and precise identification of wheat rust, facilitating timely intervention during early disease stages and thereby mitigating crop loss risks for agricultural practitioners. The proposed TGNN addresses the challenge of incomplete trust relationships among wheat pathologists by employing multi-path trust propagation with learnable weights, thereby enhancing the robustness of group decision-making, fostering efficient wheat pathologist collaboration, and ensuring the scientific rigor and reliability of disease control strategies.

The introduced overlapping community detection method, which combines the Egret Optimization Algorithm with clustering techniques, accurately captures the diverse expertise within the wheat pathologist network, uncovers complementary knowledge structures, optimizes resource allocation, and improves decision-making efficiency. Additionally, the optimization-based opinion adjustment framework effectively balances fairness and cost-efficiency within complex and uncertain group environments, providing a robust approach to achieving high-quality consensus. The integrated application of these methodologies not only elevates the quality of wheat stripe rust management decisions but also establishes a scalable technological paradigm for the intelligent advancement of agriculture.

Although the methods presented in this study demonstrate significant theoretical and practical contributions to wheat stripe rust management, some limitations remain. Firstly, the model relies heavily on the quality and consistency of multi-regional images, and low-quality images or label noise may affect diagnostic accuracy. Secondly, while the multi-path trust propagation mechanism enhances collaboration among experts, the dynamic nature of trust relationships and more complex interaction patterns have not been fully considered, limiting the flexibility of group decision-making. Additionally, the current approach focuses mainly on wheat stripe rust and lacks generalizability to other crop diseases, which restricts its broader application in agricultural disease management.

Future research could focus on several directions: (1) optimizing data preprocessing and augmentation techniques to improve model robustness against varying data quality, thereby enhancing diagnostic stability and accuracy; (2) introducing dynamic trust models and more sophisticated expert network mechanisms to boost the adaptability and intelligence of group decision-making; (3) extending the methodology to cover a wider range of crops and diseases, achieving diversification and intelligence in AI-assisted agricultural disease management; (4) integrating IoT, remote sensing, and big data technologies to develop real-time monitoring and early warning systems that promote closed-loop precision agriculture management; (5) strengthening model interpretability to increase transparency of agricultural decisions and build farmers’ trust, laying a solid foundation for the broad adoption of intelligent agriculture.

## Figures and Tables

**Table 1:** The opinion adjustment cost for differentrroles.

$e_{1}$	$e_{2}$	$e_{3}$	$e_{4}$	$e_{5}$	$e_{6}$	$e_{7}$	$e_{8}$	$e_{9}$	$e_{10}$
27	61	88	33	40	30	85	80	63	42
$e_{11}$	$e_{12}$	$e_{13}$	$e_{14}$	$e_{15}$	$e_{16}$	$e_{17}$	$e_{18}$	$e_{19}$	$e_{20}$
100	48	68	97	63	37	68	72	38	30

**Table 2:** Visualization of the trust matrix.

The Initial Trust Matrix	The Final Trust Matrix
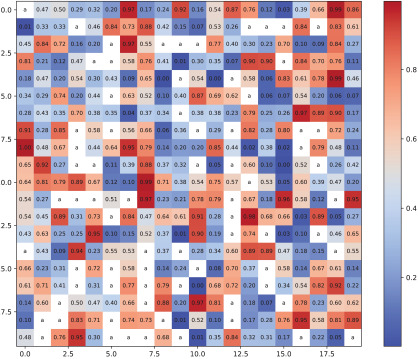	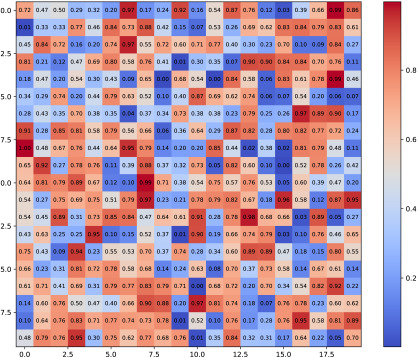

**Table 3:** Initial consensus degrees for 20 wheat pathologists across different community divisions.

3	4	5	6	7	8	9	10
0.3211	0.2945	0.2800	0.2801	0.2833	0.2426	0.2475	0.2634

**Table 4:** Overlapping community detection results.

Community	Members
$community_{1}$	$e_{3},e_{5},e_{9},e_{13},e_{15},e_{19}$
$community_{2}$	$e_{3},e_{5},e_{6},e_{7},e_{9},e_{11},e_{13},e_{15},e_{16},e_{17},e_{19}$
$community_{3}$	$e_{0},e_{1},e_{2},e_{4},e_{6},e_{8},e_{10},e_{12},e_{14},e_{16},e_{17},e_{18}$

**Table 5:** Opinion adjustment costs for different roles.

$e_{1}$	$e_{2}$	$e_{3}$	$e_{4}$	$e_{5}$	$e_{6}$	$e_{7}$	$e_{8}$	$e_{9}$	$e_{10}$
0.0366	0.0466	0.0410	0.0584	0.0455	0.0613	0.0578	0.0416	0.0408	0.0555
$e_{11}$	$e_{12}$	$e_{13}$	$e_{14}$	$e_{15}$	$e_{16}$	$e_{17}$	$e_{18}$	$e_{19}$	$e_{20}$
0.0448	0.0505	0.401	0.0567	0.0417	0.0589	0.0557	0.0535	0.0482	0.0618

**Table 6:** Weights of the three overlapping communities.

**Community**	$community_{1}$	$community_{2}$	$community_{3}$
**Weight**	0.2320	0.4026	0.3654

**Table 7:** Comparison of detection methods for crop disease diagnosis.

Manual Inspection	Experts observe symptoms in the field	Low cost, simple operation	Subjective, low efficiency, not scalable	Small-scale farms, early-stage diagnosis
Image Recognition	Capture leaf images and extract visual features	Intuitive, automated, easy to deploy	Affected by lighting and background	Single-crop disease detection, greenhouses
Sensor Technology	Monitor temperature, humidity, gas, etc.	Real-time monitoring, early warning	High cost, complex maintenance	Field environment monitoring
AI/ML Methods	Use models like CNN for image/data analysis	High accuracy, adaptive, capable of learning	Requires large data, high computational cost	Multi-crop, intelligent diagnosis systems
Remote Sensing/UAV	Satellite or drone-based large-scale imaging	Wide coverage, fast detection	Expensive, weather-dependent, limited resolution	Large-scale farms, disease spread tracking

**Table 8:** The evaluation of key attributes in crop disease detection methods.

Method	Low Cost	Easy to Deploy	Real-Time Monitoring	High Accuracy	Weather Dependency
Manual Inspection	Yes	Yes	No	No	No
Image Recognition [47]	Yes	Yes	No	Yes	No
Sensor Technology [48,49]	No	No	Yes	No	No
AI/ML Methods [50,51]	No	No	No	Yes	No
Remote Sensing/UAV [52,53]	No	No	Yes	Yes	Yes
Our Method	Yes	Yes	No	Yes	No

**Table 9:** The step-wise importance comparison in overlapping community detection.

Methods	Detecting Overlapping Communities?	The Average Community Consensus Degree	The Clustering Time Consumption	The Total Execution Time
m0	Yes	0.9517	0.0402	0.0981
m1	No	0.759	0.0115	0.1464
m2	No	0.08400	0.1502	0.1965
m3	No	0.8100	0.0295	0.0843
m4	No	0.8377	0.0324	0.0747
m5	Yes	0.8870	0.1315	0.1867
m6	Yes	0.8852	0.0452	0.1425
m7	Yes	0.8648	0.0424	0.1407
m8	Yes	0.9133	0.0812	0.1936

**Table 10:** The comparative analysis with existing methods.

Methods	Indicators	Robust Group	The Final Group Consensus	The First Adjustment Round	The Second Adjustment Round
Xu et al. [27]	-	No	0.9047	3	7
Hou et al. [58]	-	No	0.9080	5	2
Meng et al. [59]	Fairness	No	0.9215	2	4
Meng et al. [60]	Fairness and Cost	No	0.9100	2	1
Our Method	Fairness and Cost	Yes	0.9311	0	0

**Table 11:** Sensitivity analysis on the degree of missing trust values.

Degree of Missing Trust Values	The Final Ranking
70%	Plot1 ≻Plot4≻Plot2≻Plot3
50%	Plot1≻Plot4≻Plot2≻Plot3
30%	Plot1≻Plot4≻Plot2≻Plot3

**Table 12:** The sensitivity analysis of γ.

The Value of γ	The Final Ranking
0.5	Plot1≻Plot4≻Plot2≻Plot3
1	Plot1≻Plot4≻Plot2≻Plot3
2	Plot1≻Plot4≻Plot2≻Plot3
3	Plot1≻Plot4≻Plot2≻Plot3

**Table 13:** The sensitivity analysis of *k*.

The Value of *k*	The Final Ranking
3	Plot1≻Plot4≻Plot2≻Plot3
4	Plot1≻Plot4≻Plot2≻Plot3
5	Plot1≻Plot4≻Plot2≻Plot3
6	Plot1≻Plot4≻Plot2≻Plot3

**Table 14:** The sensitivity analysis of UB.

The Value of LB	The Value of UB	The Final Ranking
0.43	0.48	Plot1≻Plot4≻Plot2≻Plot3
0.43	0.96	Plot1≻Plot4≻Plot2≻Plot3
0.43	1.92	Plot1≻Plot4≻Plot2≻Plot3

**Table 15:** The sensitivity analysis of LB.

The Value of LB	The Value of UB	The Final Ranking
0.21	0.96	Plot1≻Plot4≻Plot2≻Plot3
0.43	0.96	Plot1≻Plot4≻Plot2≻Plot3
0.86	0.96	Plot1≻Plot4≻Plot2≻Plot3

## Data Availability

This study’s dataset is available at https://github.com/hihi-cpu/WheatStripRust.git (accessed on 4 May 2025). If you wish to use this dataset, please download it yourself and carefully read the usage instructions and cite the corresponding references.

## Appendix A. The Original Data

### Appendix A.1. The Wheat Pathologists’ Original Opinion Matrices

$e_{1}=\left[\begin{array}{ccccc} 21.00 & 5.06 & 2.99 & 3.94 & 4.42\\ 25.15 & 4.95 & 2.81 & 3.14 & 4.50\\ 21.67 & 4.12 & 2.42 & 2.84 & 3.71\\ 22.50 & 4.00 & 2.50 & 2.50 & 4.00 \end{array}\right]$	$e_{2}=\left[\begin{array}{ccccc} 22.91 & 5.57 & 3.36 & 4.14 & 4.96\\ 27.12 & 5.56 & 3.07 & 3.44 & 5.09\\ 23.34 & 4.46 & 2.53 & 2.99 & 4.12\\ 26.17 & 5.00 & 2.67 & 2.67 & 5.00 \end{array}\right]$
$e_{3}=\left[\begin{array}{ccccc} 19.98 & 4.62 & 2.70 & 3.61 & 4.04\\ 24.44 & 4.68 & 2.56 & 3.00 & 4.19\\ 21.04 & 4.03 & 2.30 & 2.60 & 3.62\\ 20.50 & 4.00 & 2.17 & 2.17 & 3.67 \end{array}\right]$	$e_{4}=\left[\begin{array}{ccccc} 21.75 & 5.04 & 2.95 & 3.83 & 4.39\\ 26.54 & 5.08 & 2.86 & 3.17 & 4.60\\ 23.7 & 4.46 & 2.6 & 2.89 & 4.07\\ 29.0 & 5.83 & 3.50 & 3.50 & 5.83 \end{array}\right]$
$e_{5}=\left[\begin{array}{ccccc} 22.51 & 5.38 & 3.23 & 4.01 & 4.76\\ 27.43 & 5.45 & 2.99 & 3.33 & 4.91\\ 22.41 & 4.15 & 2.36 & 2.80 & 3.75\\ 23.83 & 4.0 & 2.17 & 2.17 & 3.67 \end{array}\right]$	$e_{6}=\left[\begin{array}{ccccc} 22.00 & 5.16 & 3.04 & 3.77 & 4.53\\ 25.54 & 4.88 & 2.73 & 3.10 & 4.47\\ 24.23 & 4.81 & 2.69 & 3.06 & 4.40\\ 31.17 & 6.83 & 3.67 & 3.67 & 6.50 \end{array}\right]$
$e_{7}=\left[\begin{array}{ccccc} 21.36 & 5.12 & 2.96 & 3.82 & 4.47\\ 26.79 & 5.19 & 2.95 & 3.28 & 4.71\\ 23.51 & 4.49 & 2.65 & 3.02 & 4.08\\ 25.00 & 5.00 & 2.67 & 2.67 & 4.67 \end{array}\right]$	$e_{8}=\left[\begin{array}{ccccc} 20.99 & 4.75 & 2.76 & 3.64 & 4.14\\ 26.23 & 5.10 & 2.80 & 3.15 & 4.60\\ 21.78 & 3.85 & 2.19 & 2.58 & 3.50\\ 27.66 & 5.33 & 2.67 & 2.67 & 5.00 \end{array}\right]$
$e_{9}=\left[\begin{array}{ccccc} 22.56 & 5.34 & 3.18 & 4.05 & 4.72\\ 26.72 & 5.19 & 2.84 & 3.21 & 4.73\\ 22.98 & 4.38 & 2.60 & 2.94 & 4.07\\ 19.00 & 3.00 & 2.00 & 2.00 & 3.33 \end{array}\right]$	$e_{10}=\left[\begin{array}{ccccc} 22.03 & 5.16 & 3.04 & 3.87 & 4.53\\ 26.13 & 5.01 & 2.74 & 3.12 & 4.56\\ 22.77 & 4.31 & 2.49 & 2.79 & 3.88\\ 32.50 & 7.00 & 3.83 & 3.83 & 6.50 \end{array}\right]$
$e_{11}=\left[\begin{array}{ccccc} 20.81 & 4.86 & 2.87 & 3.64 & 4.23\\ 26.83 & 5.37 & 3.01 & 3.30 & 4.93\\ 21.87 & 3.93 & 2.22 & 2.60 & 3.54\\ 23.00 & 4.17 & 2.67 & 2.67 & 4.17 \end{array}\right]$	$e_{12}=\left[\begin{array}{ccccc} 22.54 & 5.25 & 3.16 & 3.99 & 4.67\\ 26.94 & 5.28 & 2.92 & 3.25 & 4.81\\ 24.20 & 4.81 & 2.71 & 3.11 & 4.38\\ 29.50 & 5.83 & 2.83 & 2.83 & 5.50 \end{array}\right]$
$e_{13}=\left[\begin{array}{ccccc} 22.82 & 5.32 & 3.17 & 4.02 & 4.65\\ 25.36 & 4.93 & 2.73 & 3.06 & 4.48\\ 23.56 & 4.32 & 2.51 & 2.9 & 3.93\\ 21.17 & 4.00 & 2.17 & 2.17 & 3.67 \end{array}\right]$	$e_{14}=\left[\begin{array}{ccccc} 20.67 & 4.82 & 2.78 & 3.61 & 4.18\\ 26.34 & 5.14 & 2.84 & 3.18 & 4.61\\ 23.29 & 4.48 & 2.55 & 2.83 & 4.06\\ 32.33 & 6.83 & 3.50 & 3.50 & 6.50 \end{array}\right]$
$e_{15}=\left[\begin{array}{ccccc} 22.11 & 5.19 & 3.07 & 3.93 & 4.57\\ 26.47 & 5.14 & 2.84 & 3.22 & 4.70\\ 22.17 & 4.19 & 2.49 & 2.77 & 3.83\\ 21.5 & 4.00 & 2.17 & 2.17 & 3.67 \end{array}\right]$	$e_{16}=\left[\begin{array}{ccccc} 22.05 & 5.11 & 3.03 & 3.82 & 4.54\\ 25.78 & 4.95 & 2.77 & 3.12 & 4.43\\ 22.80 & 4.23 & 2.36 & 2.72 & 3.85\\ 30.33 & 5.83 & 2.83 & 2.83 & 5.50 \end{array}\right]$
$e_{17}=\left[\begin{array}{ccccc} 22.29 & 5.29 & 3.11 & 3.90 & 4.66\\ 26.73 & 5.09 & 2.89 & 3.27 & 4.65\\ 22.33 & 4.20 & 2.44 & 2.82 & 3.77\\ 23.67 & 4.67 & 2.50 & 2.50 & 4.33 \end{array}\right]$	$e_{18}=\left[\begin{array}{ccccc} 22.13 & 5.24 & 3.11 & 3.83 & 4.60\\ 26.28 & 5.14 & 2.87 & 3.16 & 4.69\\ 23.80 & 4.42 & 2.64 & 2.96 & 4.03\\ 28.67 & 5.50 & 2.67 & 2.67 & 5.17 \end{array}\right]$

$e_{19}=\left[\begin{array}{ccccc} 21.33 & 5.01 & 2.98 & 3.87 & 4.40\\ 27.09 & 5.35 & 2.93 & 3.28 & 4.85\\ 23.03 & 4.48 & 2.55 & 2.94 & 4.09\\ 22.67 & 4.00 & 2.17 & 2.17 & 3.67 \end{array}\right]$	$e_{20}=\left[\begin{array}{ccccc} 21.85 & 5.10 & 3.04 & 3.95 & 4.49\\ 26.10 & 4.96 & 2.74 & 3.07 & 4.47\\ 21.69 & 3.91 & 2.20 & 2.58 & 3.55\\ 31.33 & 6.83 & 3.67 & 3.67 & 6.50 \end{array}\right]$

### Appendix A.2. The Original Matrices

$e_{1}=\left[\begin{array}{ccccc} 0.2100 & 0.5060 & 0.5980 & 0.7880 & 0.4911\\ 0.2515 & 0.4950 & 0.5620 & 0.6280 & 0.5000\\ 0.2167 & 0.4120 & 0.4840 & 0.5680 & 0.4122\\ 0.2250 & 0.4000 & 0.5000 & 0.5000 & 0.4444 \end{array}\right]$	$e_{2}=\left[\begin{array}{ccccc} 0.2291 & 0.5570 & 0.6720 & 0.8280 & 0.5511\\ 0.2712 & 0.5560 & 0.6140 & 0.6880 & 0.5656\\ 0.2334 & 0.4460 & 0.5060 & 0.5980 & 0.4578\\ 0.2617 & 0.5000 & 0.5333 & 0.5333 & 0.5556 \end{array}\right]$
$e_{3}=\left[\begin{array}{ccccc} 0.1998 & 0.4620 & 0.5400 & 0.7220 & 0.4489\\ 0.2443 & 0.4680 & 0.5120 & 0.6000 & 0.4656\\ 0.2104 & 0.4030 & 0.4600 & 0.5200 & 0.4022\\ 0.2050 & 0.4000 & 0.4333 & 0.4333 & 0.4074 \end{array}\right]$	$e_{4}=\left[\begin{array}{ccccc} 0.2175 & 0.5040 & 0.5900 & 0.7660 & 0.4878\\ 0.2654 & 0.5080 & 0.5720 & 0.6340 & 0.5111\\ 0.2370 & 0.4460 & 0.5200 & 0.5780 & 0.4522\\ 0.2900 & 0.5833 & 0.7000 & 0.7000 & 0.6481 \end{array}\right]$
$e_{5}=\left[\begin{array}{ccccc} 0.2251 & 0.5380 & 0.6460 & 0.8020 & 0.5289\\ 0.2743 & 0.5450 & 0.5980 & 0.6660 & 0.5456\\ 0.2241 & 0.4150 & 0.4720 & 0.5600 & 0.4167\\ 0.2383 & 0.4000 & 0.4333 & 0.4333 & 0.4074 \end{array}\right]$	$e_{6}=\left[\begin{array}{ccccc} 0.2200 & 0.5160 & 0.6080 & 0.7540 & 0.5033\\ 0.2554 & 0.4880 & 0.5460 & 0.6200 & 0.4967\\ 0.2423 & 0.4810 & 0.5380 & 0.6120 & 0.4889\\ 0.3117 & 0.6833 & 0.7333 & 0.7333 & 0.7222 \end{array}\right]$
$e_{7}=\left[\begin{array}{ccccc} 0.2136 & 0.5120 & 0.5920 & 0.7640 & 0.4967\\ 0.2678 & 0.5190 & 0.5900 & 0.6560 & 0.5233\\ 0.2351 & 0.4490 & 0.5300 & 0.6040 & 0.4533\\ 0.2500 & 0.5000 & 0.5333 & 0.5333 & 0.5185 \end{array}\right]$	$e_{8}=\left[\begin{array}{ccccc} 0.2099 & 0.4750 & 0.5520 & 0.7280 & 0.4600\\ 0.2623 & 0.5100 & 0.5600 & 0.6300 & 0.5111\\ 0.2178 & 0.3850 & 0.4380 & 0.5160 & 0.3889\\ 0.2767 & 0.5333 & 0.5333 & 0.5333 & 0.5556 \end{array}\right]$
$e_{9}=\left[\begin{array}{ccccc} 0.2256 & 0.5340 & 0.6360 & 0.8100 & 0.5244\\ 0.2672 & 0.5190 & 0.5680 & 0.6420 & 0.5256\\ 0.2298 & 0.4380 & 0.5200 & 0.5880 & 0.4522\\ 0.1900 & 0.3000 & 0.4000 & 0.4000 & 0.3704 \end{array}\right]$	$e_{10}=\left[\begin{array}{ccccc} 0.2203 & 0.5160 & 0.6080 & 0.7740 & 0.5033\\ 0.2613 & 0.5010 & 0.5480 & 0.6240 & 0.5067\\ 0.2277 & 0.4310 & 0.4980 & 0.5580 & 0.4311\\ 0.3250 & 0.7000 & 0.7667 & 0.7667 & 0.7222 \end{array}\right]$
$e_{11}=\left[\begin{array}{ccccc} 0.2081 & 0.4860 & 0.5740 & 0.7280 & 0.4700\\ 0.2683 & 0.5370 & 0.6020 & 0.6600 & 0.5478\\ 0.2187 & 0.3930 & 0.4440 & 0.5200 & 0.3933\\ 0.2300 & 0.4167 & 0.5333 & 0.5333 & 0.4630 \end{array}\right]$	$e_{12}=\left[\begin{array}{ccccc} 0.2254 & 0.5250 & 0.6320 & 0.7980 & 0.5189\\ 0.2694 & 0.5280 & 0.5840 & 0.6500 & 0.5344\\ 0.2420 & 0.4810 & 0.5420 & 0.6220 & 0.4867\\ 0.2950 & 0.5833 & 0.5667 & 0.5667 & 0.6111 \end{array}\right]$
$e_{13}=\left[\begin{array}{ccccc} 0.2282 & 0.5320 & 0.6340 & 0.8040 & 0.5167\\ 0.2536 & 0.4930 & 0.5460 & 0.6120 & 0.4978\\ 0.2356 & 0.4320 & 0.5020 & 0.5800 & 0.4367\\ 0.2117 & 0.4000 & 0.4333 & 0.4333 & 0.4074 \end{array}\right]$	$e_{14}=\left[\begin{array}{ccccc} 0.2067 & 0.4820 & 0.5560 & 0.7220 & 0.4644\\ 0.2634 & 0.5140 & 0.5680 & 0.6360 & 0.5122\\ 0.2329 & 0.4480 & 0.5100 & 0.5660 & 0.4511\\ 0.3233 & 0.6833 & 0.7000 & 0.7000 & 0.7222 \end{array}\right]$
$e_{15}=\left[\begin{array}{ccccc} 0.2211 & 0.5190 & 0.6140 & 0.7860 & 0.5078\\ 0.2647 & 0.5140 & 0.5680 & 0.6440 & 0.5222\\ 0.2217 & 0.4190 & 0.4980 & 0.5540 & 0.4256\\ 0.2150 & 0.4000 & 0.4333 & 0.4333 & 0.4074 \end{array}\right]$	$e_{16}=\left[\begin{array}{ccccc} 0.2205 & 0.5110 & 0.6060 & 0.7640 & 0.5044\\ 0.2578 & 0.4950 & 0.5540 & 0.6240 & 0.4922\\ 0.2280 & 0.4230 & 0.4720 & 0.5440 & 0.4278\\ 0.3033 & 0.5833 & 0.5667 & 0.5667 & 0.6111 \end{array}\right]$
$e_{17}=\left[\begin{array}{ccccc} 0.2229 & 0.5290 & 0.6220 & 0.7800 & 0.5178\\ 0.2673 & 0.5090 & 0.5780 & 0.6540 & 0.5167\\ 0.2233 & 0.4200 & 0.4880 & 0.5640 & 0.4189\\ 0.2367 & 0.4667 & 0.5000 & 0.5000 & 0.4815 \end{array}\right]$	$e_{18}=\left[\begin{array}{ccccc} 0.2213 & 0.5240 & 0.6220 & 0.7660 & 0.5111\\ 0.2628 & 0.5140 & 0.5740 & 0.6320 & 0.5211\\ 0.2380 & 0.4420 & 0.5280 & 0.5920 & 0.4478\\ 0.2867 & 0.5500 & 0.5333 & 0.5333 & 0.5741 \end{array}\right]$
$e_{19}=\left[\begin{array}{ccccc} 0.2133 & 0.5010 & 0.5960 & 0.7740 & 0.4889\\ 0.2709 & 0.5350 & 0.5860 & 0.6560 & 0.5389\\ 0.2303 & 0.4480 & 0.5100 & 0.5880 & 0.4544\\ 0.2267 & 0.4000 & 0.4333 & 0.4333 & 0.4074 \end{array}\right]$	$e_{20}=\left[\begin{array}{ccccc} 0.2185 & 0.5100 & 0.6080 & 0.7900 & 0.4989\\ 0.2610 & 0.4960 & 0.5480 & 0.6140 & 0.4967\\ 0.2169 & 0.3910 & 0.4400 & 0.5160 & 0.3944\\ 0.3133 & 0.6833 & 0.7333 & 0.7333 & 0.7222 \end{array}\right]$

## Appendix B. Group Opinions After the First-Stage Processing

$community_{1}=\left[\begin{array}{ccccc} 0.2172 & 0.5065 & 0.5960 & 0.7617 & 0.4937\\ 0.2607 & 0.5003 & 0.5560 & 0.6253 & 0.5026\\ 0.2308 & 0.4367 & 0.4963 & 0.5623 & 0.4409\\ 0.3111 & 0.6527 & 0.7000 & 0.7000 & 0.6913 \end{array}\right]$
$community_{2}=\left[\begin{array}{ccccc} 0.2179 & 0.5095 & 0.5996 & 0.7642 & 0.4970\\ 0.2631 & 0.5075 & 0.5656 & 0.6340 & 0.5111\\ 0.2310 & 0.4361 & 0.5004 & 0.5702 & 0.4401\\ 0.2920 & 0.5954 & 0.6242 & 0.6242 & 0.6263 \end{array}\right]$
$community_{3}=\left[\begin{array}{ccccc} 0.2182 & 0.5167 & 0.6122 & 0.7793 & 0.5045\\ 0.2637 & 0.5170 & 0.5748 & 0.6448 & 0.5225\\ 0.2264 & 0.4264 & 0.4952 & 0.5697 & 0.4309\\ 0.2314 & 0.4278 & 0.4750 & 0.4750 & 0.4537 \end{array}\right]$
